# Transcription factor Hlx controls a systematic switch from white to brown fat through Prdm16-mediated co-activation

**DOI:** 10.1038/s41467-017-00098-2

**Published:** 2017-07-12

**Authors:** Lei Huang, Dongning Pan, Qingbo Chen, Lihua J. Zhu, Jianhong Ou, Martin Wabitsch, Yong-Xu Wang

**Affiliations:** 1 Department of Molecular, Cell and Cancer Biology, Program in Molecular Medicine, University of Massachusetts Medical School, 364 Plantation Street, Worcester, MA 01605 USA; 2 Department of Pediatrics and Adolescent Medicine, Division of Pediatric Endocrinology and Diabetes, University Medical Center Ulm, Ulm, 89075 Germany

## Abstract

Browning of subcutaneous white fat (iWAT) involves several reprograming events, but the underlying mechanisms are incompletely understood. Here we show that the transcription factor Hlx is selectively expressed in brown adipose tissue (BAT) and iWAT, and is translationally upregulated by β3-adrenergic signaling-mediated suppression of the translational inhibitor 4E-BP1. Hlx interacts with and is co-activated by Prdm16 to control BAT-selective gene expression and mitochondrial biogenesis. *Hlx* heterozygous knockout mice have defects in brown-like adipocyte formation in iWAT, and develop glucose intolerance and high fat-induced hepatic steatosis. Conversely, transgenic expression of Hlx at a physiological level drives a full program of thermogenesis and converts iWAT to brown-like fat, which improves glucose homeostasis and prevents obesity and hepatic steatosis. The adipose remodeling phenotypes are recapitulated by fat-specific injection of Hlx knockdown and overexpression viruses, respectively. Our studies establish Hlx as a powerful regulator for systematic white adipose tissue browning and offer molecular insights into the underlying transcriptional mechanism.

## Abstract (web summary)

The transcriptional co-activator Prdm16 regulates browning of white adipose tissue (WAT). Here, the authors show that Prdm16 interacts with the transcription factor Hlx, which is stabilized in response to β3-adrenergic signaling, to increase thermogenic gene expression and mitochondrial biogenesis in subcutaneous WAT.

## Introduction

Classical brown adipose tissue (BAT) and brown-like adipocytes (also named beige or brite adipocytes) are specialized for energy expenditure by dissipating energy as heat in a process called nonshivering thermogenesis^1–4^. In rodents, while BAT depots primarily exist in the interscapular region; beige adipocytes, often present as small clusters, are dispersed within certain WAT depots, especially iWAT, and are readily induced by catecholamines, cold, and other external cues^5–9^. Studies in recent years demonstrate that adult humans do have metabolically active BAT depots^10–13^ that are heterogeneous, comprising both classical brown adipocytes and beige adipocytes^14–19^. Moreover, it appears that human BAT mass is inversely associated with body mass index^10, 12^ and age^10, 20^, and can be recruited after cold exposure^12, 21–23^. These findings have led to significant interest in developing strategies to promote the activity of BAT or to convert white adipose tissue (WAT) to brown-like fat as potential therapeutic avenues for obesity and metabolic diseases^24^.

Similar to BAT, beige adipocytes possess, albeit in a less degree, several prominent features that are indispensable elements for a full program of thermogenesis and effective energy dissipation. These adipocytes have a multilocular lipid droplet morphology, express a set of thermogenic genes, including Ucp1, which uncouples oxidative phosphorylation from ATP production to generate heat, and have densely packed mitochondria as well as considerable vasculature and innervation^1–4^. Clearly, several distinct processes are involved in beige adipocyte development or browning of WAT, but how they are executed in a concerted manner is not well understood. Moreover, while a variety of agents and genetic manipulations have been shown to induce browning of WAT^2, 3, 25^, a comprehensive molecular characterization of the browned WAT depots has not been performed, and to what extent the browning has occurred has been unclear in most of these studies. From a therapeutic point of view, this is not a trivial issue, as a robust browning of WAT is presumably a preferred goal.

Transcriptional co-activator Prdm16 has been demonstrated to play a central role in browning of WAT^26–29^; however, its mechanism of action and the involved transcription factor(s) remain to be fully elucidated. Here we show that transcription factor Hlx, at least in part through its interaction with and co-activation by Prdm16, drives a full program of thermogenesis. Our studies identified Hlx as a major regulator for systematic and robust browning of iWAT, and may provide a potential therapeutic target for obesity and metabolic diseases.

## Results

### Translational regulation of Hlx by β3-adrenergic signaling

**Fig. 1:**
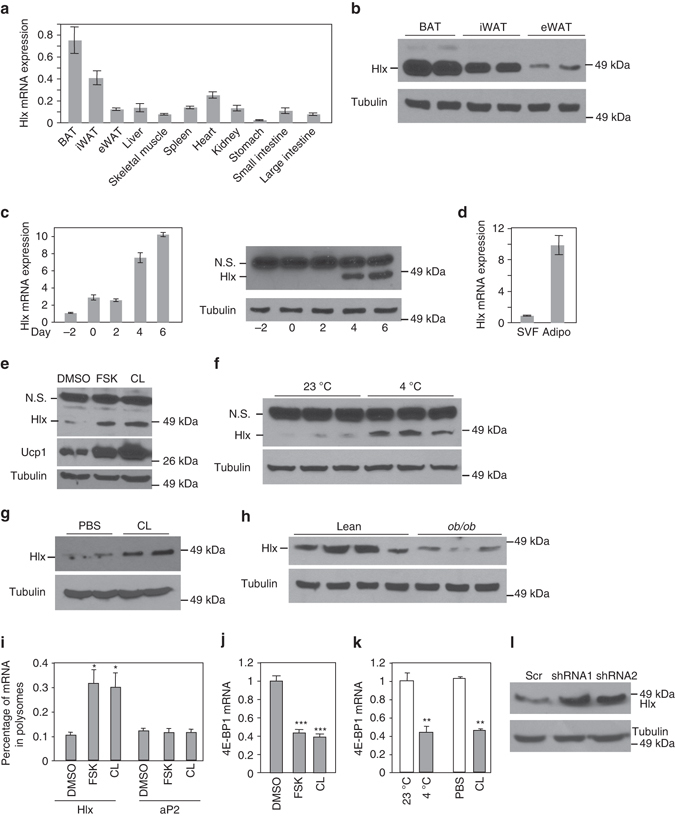
Hlx is BAT-enriched and is translationally regulated in iWAT by β3-adrenergic activation. **a** qPCR analysis of *Hlx* mRNA expression in multiple tissues from 12-week-old male mice (*n* = 4). **b** Western blot analysis of Hlx protein in different fat depots from 8-week-old male mice. **c** Hlx mRNA (*n* = 3) and protein levels at different time points of in vitro differentiation of immortalized brown preadipocytes. N.S. indicates a non-specific band. **d** *Hlx* mRNA expression in stromal vascular fraction (SVF) and mature adipocyte fraction isolated from BAT of 12-week-old male mice (*n* = 3). **e** Western blot analysis of Hlx and Ucp1 protein in in vitro differentiated brown adipocytes treated with Forskolin or CL-316,243 for 12 h. N.S. indicates a non-specific band. **f** Western blot analysis of Hlx protein in iWAT of 12-week-old male mice at room temperature or cold challenged for 6 h. N.S. indicates a non-specific band. **g** Western blot analysis of Hlx protein in iWAT of 12-week-old male mice. Mice were intraperitoneally injected with a single dose of CL-316,243 or PBS, and were sacrificed 24 h after injection. **h** Western blot analysis of Hlx protein in iWAT of 3-month-old *ob/ob* mice and C57BL6 mice. **i** Polysome analysis. Brown adipocytes treated with Forskolin or CL-316,243 for 9 h (*n* = 3). Percentage of *Hlx* mRNA associated with ployribosome was determined by RT-qPCR. **j** *4E-BP1* mRNA expression in brown adipocytes treated with Forskolin or CL-316,243 for 9 h (*n* = 4). **k** *4E-BP1* mRNA expression in iWAT of 3-month-old male mice cold challenged for 6 h (*n* = 4 mice per group) or injected with a single dose of CL-316,243 (*n* = 3 mice per group). **l** Western blot analysis of Hlx protein in brown adipocytes expressing 4E-BP1 knockdown lentiviruses. All error bars represent s.e.m. Two-tailed unpaired Student’s t-test was performed. *p < 0.05; **p < 0.01; ***p < 0.001

BAT-enriched transcription factors have the potential to be involved in brown and beige adipocyte development/maintenance. To identify such candidates, we analyzed our published RNA-Seq datasets of mouse BAT and epididymal WAT (eWAT)^30^, and found that the nonclustered H2.0-like homeobox (*Hlx*) gene was highly enriched in BAT vs. eWAT with mRNA levels of 16.8 FPKM and 1.5 FPKM, respectively. Real time-quantitative PCR (RT-qPCR) analysis showed that BAT had the highest expression of *Hlx*, followed by iWAT, whereas other tissues expressed a relatively low amount (Fig. 1a). Similarly, Hlx protein displayed a pattern of BAT > iWAT > eWAT (Fig. 1b), mirroring Ucp1 expression. Hlx was induced during adipogenesis, evidenced by both brown-cell differentiation in vitro and fractionated adipose tissue (Figs. 1c, d).

We examined whether Hlx level is regulated by β3-adrenergic signaling. Treatment of mature brown adipocytes in vitro with a specific β3-adrenergic receptor agonist CL-316,243 or an activator of its downstream enzyme adenylyl cyclase, Forskolin, elevated Hlx protein level (Fig. 1e), but surprisingly, had no effect on its mRNA expression (Supplementary Fig. 1a). Acute cold challenging of mice at 4 °C for 6  hr increased Hlx protein but not its mRNA level in iWAT (Fig. 1f and Supplementary Fig. 1b); however, no change was found in BAT (Supplementary Fig. 1c), likely due to the presence of a considerable basal level of constitutive β3-adrenergic activation. Similar results were obtained with a single-dose intraperitoneal injection of CL-316,243 (Fig. 1g and Supplementary Fig. 1d). β3-adrenergic receptor function is severely impaired in WAT of *ob/ob* mice^31^. Consistent with this, Hlx protein, but not its mRNA, was markedly decreased in iWAT of this mouse model (Fig. 1h and Supplementary Fig. 1e). We compared the half time of Hlx protein with and without CL-316,243 in cultured adipocytes, and observed no difference (Supplementary Fig. 1f), indicating that Hlx is not regulated at post-translational level. We next performed polysome analysis of cultured adipocytes. RT-qPCR examination of RNA samples prepared from sucrose gradient fractions revealed that *Hlx* mRNA, but not *aP2* mRNA, was present with a significantly higher percentage in the actively translated, polyribosome fractions when adipocytes were treated with Forskolin or CL-316,243 (Fig. 1i), suggesting an elevated *Hlx* mRNA translation efficiency. Mice lacking the translational inhibitor 4E-BP1 show browning of WAT^32^. We found that β3-adrenergic activation suppressed the expression of *4E-BP1* both in adipocytes in vitro and adipose tissue in vivo (Figs. 1j, k). Moreover, knockdown of 4E-BP1 increased Hlx protein level (Fig. 1l and Supplementary Fig. 1g). These results suggest that β3-adrenergic activation promotes Hlx translation through suppressing *4E-BP1* expression. Overall, the selective expression profile of Hlx and the unexpected translational regulation indicate a potentially important role of Hlx in brown adipocytes and browning of WAT.

### Hlx induces BAT gene expression and mitochondrial biogenesis

**Fig. 2:**
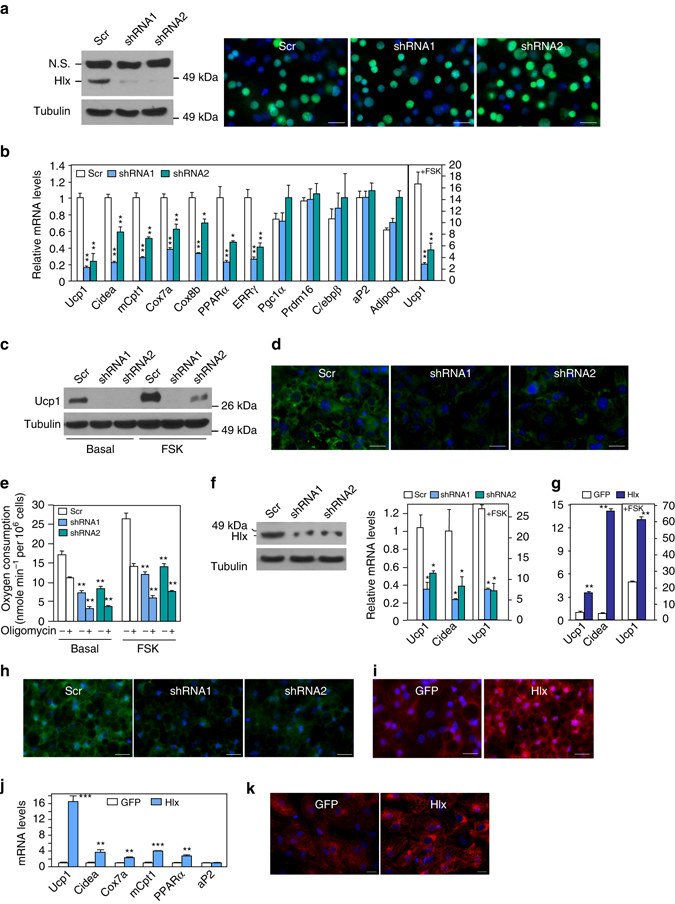
Hlx regulates thermogenic gene expression and mitochondrial biogenesis in adipocyte culture. **a** Brown preadipocytes were infected with Hlx knockdown lentiviruses and differentiated. Western blot analysis of Hlx protein and immunostaining of C/ebpα were performed. *Green*, C/ebpα; *blue*, DAPI. *Scale bar*, 200 µm. N.S. indicates a nonspecific band. **b** Brown adipocytes generated as in (**a**) were either untreated or treated with Forskolin for 6 h, and gene expression was analyzed (*n* = 3). **c** Brown adipocytes generated as in (**a**) were either untreated or treated with Forskolin for 6 h, and Ucp1 protein was analyzed. **d** Brown adipocytes generated as in (**a**) were stained with a Tom20 antibody. Shown are representative images of three experiments. *Green*, Tom20; *blue*, DAPI. *Scale bar*, 200 µm. **e** Brown adipocytes generated as in (**a**) were either untreated or treated with Forskolin for 12 h. Oxygen consumption was measured with a Clark-type electrode without or with Oligomycin A (1 µM) (*n* = 3). **f** Primary iWAT preadipocytes were infected with Hlx knockdown lentiviruses and differentiated. *Left*, western blot analysis of Hlx protein were performed. *Right*, adipocytes were either untreated or treated with Forskolin for 6 h, and gene expression was analyzed (*n* = 3). **g** In vitro differentiated primary iWAT adipocytes were infected with GFP or Hlx adenoviruses for 36 h. Cells were then treated with Forskolin for 6 h or left untreated, and gene expression was analyzed (*n* = 3). **h** Primary mature iWAT adipocytes generated as in (**f**) were stained with a Tom20 antibody. Shown are representative images of three experiments. *Green*, Tom20; *blue*, DAPI. Scale bar, 200 µm. **i** Primary mature iWAT adipocytes generated as in (**g**) were stained with a Tom20 antibody. Shown are representative images of three experiments. *Red*, Tom20; *blue*, DAPI. Scale bar, 200 µm. **j** Gene expression was analyzed in differentiated human adipocytes infected with adenoviruses expressing human Hlx (*n* = 3). **k** Differentiated human adipocytes infected with adenoviruses expressing human Hlx were stained with a Tom20 antibody. Shown are representative images of three experiments. *Red*, Tom20; *blue*, DAPI. Scale bar, 200 µm. All error bars represent s.e.m. Two-tailed unpaired Student’s t-test was performed. *p < 0.05; **p < 0.01; ***p < 0.001

To investigate whether Hlx is required for adipocyte function, we used lentivirus to stably express two Hlx shRNA knockdown constructs in immortalized brown preadipocytes, which were then induced to undergo adipocyte differentiation. Hlx shRNA effectively reduced Hlx expression (Fig. 2a) but did not affect adipocyte differentiation as indicated by both C/ebpα immunostaining and Oil Red O staining (Fig. 2a and Supplementary Fig. 2a) and normal expression levels of common fat genes *aP2* and *Adipoq* (Fig. 2b). Interestingly, both basal and Forskolin-induced *Ucp1* mRNA and protein were markedly reduced (Figs. 2b, c). Knockdown of Hlx also decreased the expression of *Cidea* and mitochondrial enzyme *mCpt1*, *Cox7a* and *Cox8b* (Fig. 2b). The use of two independent knockdown constructs, together with the data described below from primary *Hlx* knockout adipocytes, suggests that these are not off-target effects of shRNA. Similar results were obtained when cells were collected at different time points during the course of differentiation (Supplementary Fig. 2b). Whereas co-activator *Pgc-1α* and *Prdm16* expression remained unchanged, *Pparα* and *Errγ*, transcription factors that are primarily involved in mitochondrial biogenesis, were downregulated (Fig. 2b). These data led us to examine whether Hlx is required for mitochondrial biogenesis. Indeed, consistent with the gene expression data, knockdown of Hlx in brown adipocytes led to diminished mitochondrial marker Tom20 immunostaining and MitoTracker staining, suggesting attenuated mitochondrial formation (Fig. 2d and Supplementary Fig. 2c). Importantly, the effect of Hlx knockdown is functionally relevant, as total and uncoupled oxygen consumption rate was significantly reduced at both basal and Forskolin-stimulated conditions (Fig. 2e). FCCP-stimulated maximal respiration was also lower (Supplementary Fig. 2d).

iWAT expresses an appreciable level of Hlx. Similar to what is observed in brown adipocytes, knockdown of Hlx in primary iWAT adipocytes had no effect on adipogenesis (Supplementary Fig. 2e), but decreased *Ucp1* and *Cidea* expression and strongly suppressed mitochondrial biogenesis (Figs. 2f, h). To complement these loss-of-functional studies, we used adenovirus to acutely overexpress Hlx in differentiated primary iWAT adipocytes. Ectopic expression of Hlx increased *Ucp1* and *Cidea* expression and promoted mitochondrial biogenesis (Figs. 2g, i). Moreover, adenoviral overexpression of human ortholog of Hlx similarly induced *Ucp1* expression and mitochondrial biogenesis in human adipocytes (Figs. 2j, k). These combined results suggest that, in both mouse and human adipocytes, Hlx may govern two parallel processes, BAT-selective gene expression and mitochondrial biogenesis, without affecting adipogenesis per se, which is remarkably similar to the roles of transcriptional co-activator Prdm16^27^.

### Fat-specific Hlx knockdown and overexpression remodel iWAT

**Fig. 3:**
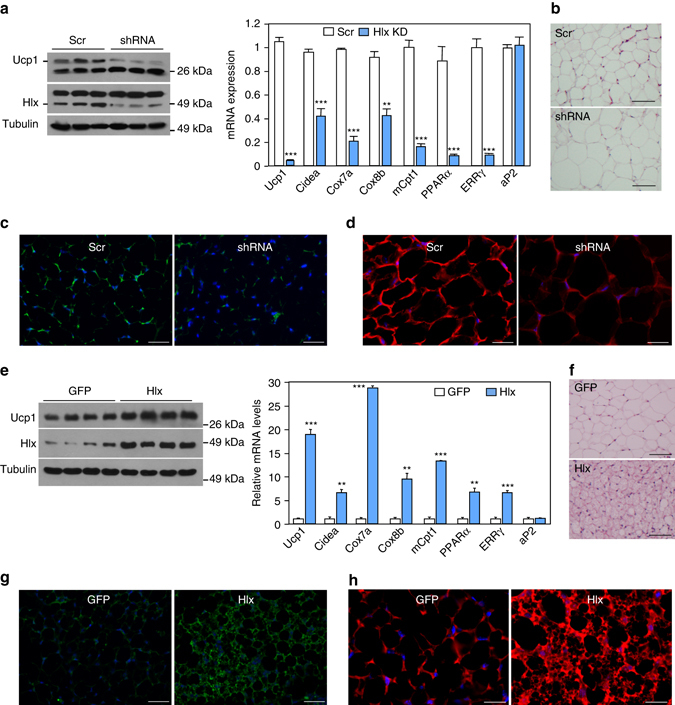
Fat-specific Hlx knockdown and overexpression through viral injection remodel WAT in vivo. **a** iWAT of wild-type mice was injected with Hlx knockdown lentiviruses. Protein and mRNA expression were analyzed (*n* = 3 mice per group). **b** H&E staining of iWAT injected with Hlx knockdown lentiviruses. Shown are representative images of three mice per group. *Scale bar*, 200 µm. **c**, **d** Ucp1 staining (**c**) and MitoTracker staining (**d**) of iWAT injected with Hlx knockdown lentiviruses. Shown are representative images of three mice per group. *Blue*, DAPI. *Scale bar*, 200 µm. **e** iWAT of wild-type mice was injected with Hlx overexpression adenoviruses. Protein and mRNA expression were analyzed (*n* = 4 mice per group). **f** H&E staining of iWAT injected with Hlx overexpression adenoviruses. Shown are representative images of three mice per group. *Scale bar*, 200 µm. **g**, **h** Ucp1 staining (**g**) and MitoTracker staining (**h**) of iWAT injected with Hlx overexpression adenoviruses. Shown are representative images of three mice per group. *Blue*, DAPI. *Scale bar*, 200 µm. All injections were repeated with independent cohorts of mice. Tom20 staining was also performed in (**d**) and (**h**) and similar results were obtained. All error bars represent s.e.m. Two-tailed unpaired Student’s t-test was performed. **p < 0.01; ***p < 0.001

We asked whether the effects of Hlx can be recapitulated in vivo. We knocked down or overexpressed Hlx in iWAT through viral injection. To rule out potential side effects of viral infection and signaling on browning, equal number of experimental and control viruses were injected into the left and right iWAT pads of the same animal, respectively. One week after initial injection, fat tissues were collected. Strikingly, samples with Hlx knockdown lentiviral injection expressed much lower levels of *Ucp1*, *Cidea*, and mitochondrial oxidative genes, compared with scramble control samples (Fig. 3a). Their adipocytes were larger and mostly Ucp1-negative, and had diminished mitochondrial staining (Figs. 3b–d), resembling a typical visceral-like WAT. Conversely, injection of Hlx adenoviruses into iWAT pads produced a brown-like phenotype, compared with control adenoviral injection of the same animal. This included a darker appearance, elevated levels of *Ucp1* and mitochondrial oxidative genes, smaller adipocytes that were almost all Ucp1-positive, and augmented mitochondrial biogenesis (Figs. 3e–h). Thus, Hlx knockdown and overexpression through viral delivery directly led to remarkable, but opposite, remodeling of iWAT in vivo. Given the fat-specific nature of the viral delivery and the short duration of the experiments (1 week), the observed phenotypes were most likely due to “transdifferentiation” of preexisting preadipocytes/adipocytes.

### *Hlx* heterozygous mice are defective in iWAT browning

**Fig. 4:**
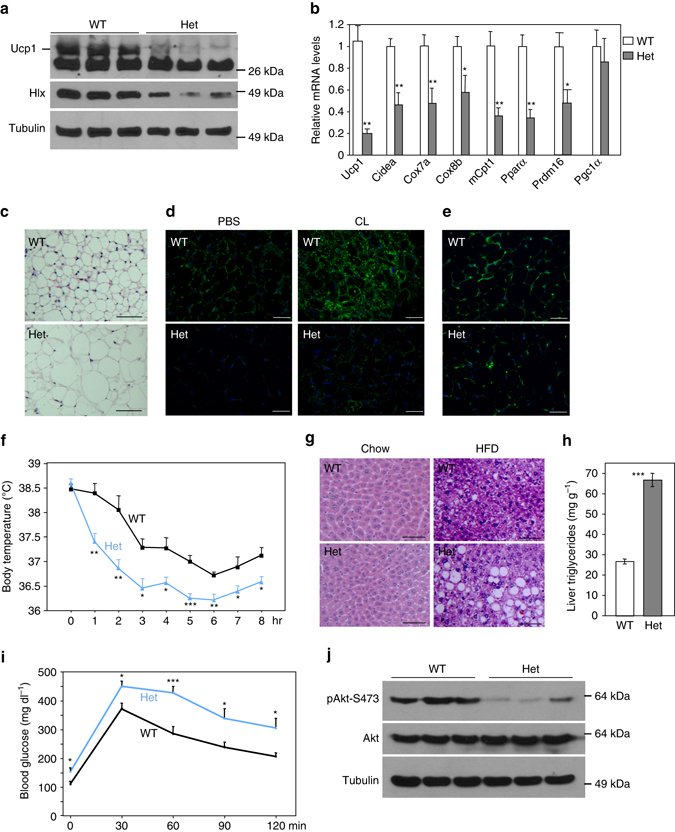
Loss of beige adipocytes, glucose intolerance and hepatic steatosis in *Hlx* heterozygous mice. **a** Western blot analysis of Ucp1 and Hlx protein in iWAT of 8-week-old male Hlx heterozygous mice and littermate controls. **b** qPCR analysis of gene expression in iWAT of 12-week-old male *Hlx* heterozygous mice (*n* = 5) and littermate controls (*n* = 7). **c** H&E staining of iWAT of 8-week-old male *Hlx* heterozygous mice and littermate controls. Shown are representative images of three mice per genotype. *Scale bar*, 200 µm. **d** Ucp1 immunofluorescence staining of iWAT of 8-week-old male *Hlx* heterozygous mice and littermate controls that has received daily CL-316243 or PBS intraperitoneal injection for 10 days. Shown are representative images of three mice per genotype. *Green*, Ucp1; *blue*, DAPI. *Scale bar*, 200 µm. **e** iWAT of 10-week-old male *Hlx* heterozygous mice and littermate controls was immunostained with a Tom20 antibody. Shown are representative images of three mice per genotype. *Green*, Tom20; *blue*, DAPI. *Scale bar*, 200 µm. **f** Body temperature of 8-month-old male *Hlx* heterozygous mice (*n* = 8) and littermate controls (*n* = 6) at 4 °C. **g** H&E staining of liver of Hlx heterozygous mice on a normal chow or on a high-fat diet for 11 weeks. Shown are representative images of three mice per genotype. **h** Hepatic triglyceride content of *Hlx* heterozygous mice and littermate controls (*n* = 3 per genotype) on a high-fat diet for 11 weeks. **i** Glucose tolerance test of 10-month-old male *Hlx* heterozygous mice and littermate controls (*n* = 7 mice per group). **j** Phosphorylation level of Akt at residue S473 in iWAT. All *error bars* represent s.e.m. Two-tailed unpaired Student’s t-test was performed. *p < 0.05; **p < 0.01; ***p < 0.001

*Hlx* homozygous null mice are embryonically lethal. To further investigate the in vivo function of Hlx in adipose tissue, we used *Hlx* heterozygous knockout mice, which are healthy and outwardly indistinguishable from wild-type littermates. Despite decreased Hlx expression (Supplementary Fig. 3a), levels of *Ucp1* and mitochondrial oxidative genes in BAT remained unchanged in Hlx heterozygous mice (Supplementary Fig. 3b, c), indicating that a single copy of *Hlx* might be sufficient to maintain normal gene expression in classical BAT. However, these genes were strongly downregulated in the iWAT at room temperature (Figs. 4a, b) and after 6-hr cold exposure (Supplementary Fig. 3d), indicating compromised beige adipocyte formation. Indeed, despite similar body weight and iWAT mass between the two genotypes, iWAT adipocytes of the heterozygous mice were much larger in size with a unilocular lipid droplet (Fig. 4c). Immunofluorescence staining revealed a great loss of Ucp1-positve adipocytes (Fig. 4d) and decreased mitochondrial biogenesis (Fig. 4e). Given that similar effects were also observed in iWAT adipocytes with Hlx knockdown both in vitro (Figs. 2f, h) and in vivo (Figs. 3b–d), this haploinsufficient effect is likely to be cell-autonomous, a notion that was strongly supported by data obtained from *Hlx* heterozygous primary iWAT adipocytes (Supplementary Fig. 3e, f). To further investigate whether the iWAT of the heterozygous mice is defective in browning, we treated mice with CL-316,243 for 10 days. Wild-type mice showed a robust induction of Ucp1-positive beige adipocytes by CL-316,243; this was strongly blocked in the heterozygous mice (Fig. 4d). Our data indicate that Hlx is critically required for beige adipocyte formation and a single copy of *Hlx* is not sufficient to activate the browning program.

### Metabolic phenotypes of *Hlx* heterozygous mice

We tested whether loss of beige adipocytes in the *Hlx* heterozygous mice leads to physiological and metabolic abnormalities. We first examined cold tolerance by placing 3-month-old mice at 4 °C. Body temperature was similar between the heterozygous mice and littermate controls during the first 4 h; however, starting from 5 h, the heterozygous mice had a significantly lower body temperature (Supplementary Fig. 3g). We have previously observed that suppression of iWAT browning renders aged mice more susceptible to cold challenge^30^. Indeed, the cold intolerance phenotype was much more evident in 8-month-old *Hlx* heterozygous mice; body temperature was significantly lower at the first 1 h time point and onward (Fig. 4f).

Food intake for either normal chow or high-fat diet was similar between the two genotypes (Supplementary Fig. 3h). We fed the heterozygous mice and littermate controls with a high-fat diet for 11 weeks. While no significant body weight difference was observed (Supplementary Fig. 3i), the heterozygous mice had a much higher triglyceride accumulation in the liver (Fig. 4g, h). Their iWAT continued to show a decreased expression of *Ucp1* and mitochondrial oxidative genes (Supplementary Fig. 3j). Since liver of the heterozygous mice had normal expression of mitochondrial oxidative genes and lipogenic genes under normal chow diet (Supplementary Fig. 3k), it is possible that the high-fat diet-induced hepatic steatosis is secondary, resulted from loss of beige adipocytes. Interestingly, high-fat diet-induced hepatic steatosis was also observed in *Prdm16* knockout mice prior to body weight difference, which occurred after 20-week high-fat diet^28^.

We assessed glucose homeostasis in *Hlx* heterozygous mice. At 3-months of age, heterozygous mice showed a trend of being less glucose tolerant, but only 30-min time point reached statistical significance. At 10-months of age, the heterozygous group had significantly higher glucose levels at both fed state (179.1 ± 11.2 mg dl^−1^ for wild type vs. 224.4 + 12.6 mg dl^−1^ for Het, *p* = 0.02, Student’s t-test) and fasting state (111.1 ± 9.0 mg dl^−1^ for wild type vs. 153.9 + 12.9 mg dl^−1^ for Het, *p* = 0.02, Student’s t-test). While no significant difference was observed in an insulin tolerance test when normalized with their initial glucose levels, the heterozygous mice had worse glucose tolerance (Fig. 4i). Levels of phosphorylated Akt remained unchanged in skeletal muscle, liver, and heart (Supplementary Fig. 3l), but were significantly decreased in iWAT (Fig. 4j). In total, loss of beige adipocytes caused by Hlx haploinsufficiency is associated with decreased body temperature at cold, increased blood glucose level, deteriorated glucose tolerance, and high-fat diet-induced hepatic steatosis.

### Transgenic expression of Hlx converts white fat to brown fat

**Fig. 5:**
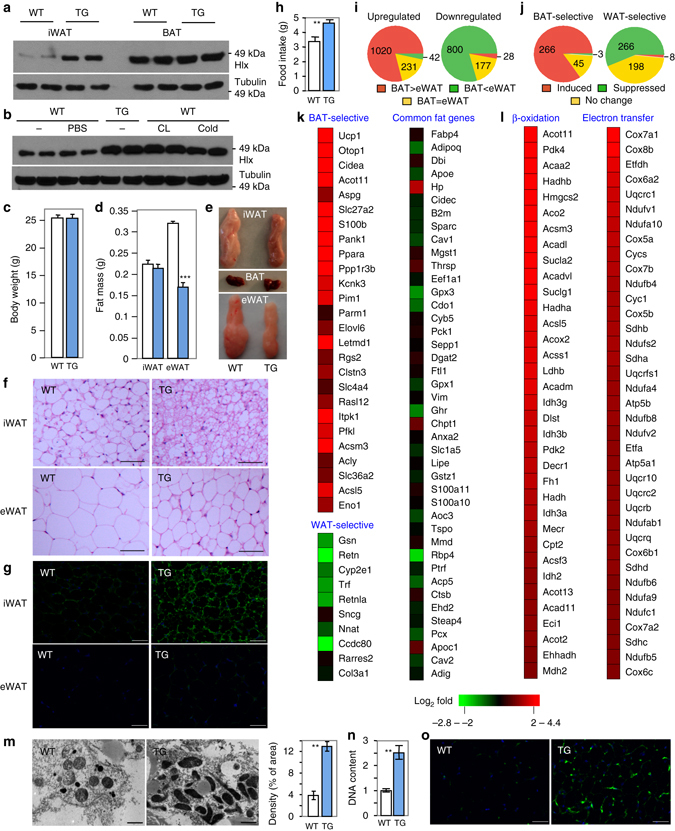
*Hlx* transgene systematically remodels iWAT to generate a brown-like fat. **a** Western blot analysis of Hlx protein in fat depots of transgenic mice and control mice. **b** Mice were intraperitoneally injected with a single dose of CL-316,243, or placed at 4 °C for 6 hr. Hlx protein levels in iWAT were compared with that of *Hlx* transgenic mice. **c** Body weights of 10-week-old male (*n* = 4–5 per group). **d** Fat mass of mice shown in (**c**). *White bar*, wild type; *Blue bar*, transgenic mice. **e** Representative images of BAT, iWAT and eWAT. **f** H&E staining of iWAT and eWAT of 12-week-old mice. Scale bar, 200 µm. **g** Ucp1 immunofluorescence staining of iWAT and eWAT of 12-week-old mice. Shown are representative images of three mice per genotype. *Green*, Ucp1; *Blue*, DAPI. *Scale bar*, 200 µm. **h** Daily food intake of 12-week-old male mice (*n* = 5 per group). **i** 1293 significantly upregulated genes (*left*) and 1005 significantly downregulated (*right*) genes were identified in the iWAT of *Hlx* transgenic mice. In wild type mice, these genes have either similar expression between BAT and eWAT, higher expression in BAT, or higher expression in eWAT. Shown are distributions of these genes. **j** Distribution of BAT-selective (*left*) and WAT-selective genes (*right*) based on how they are regulated in the iWAT by the *Hlx* transgene. **k** Heat maps of relative expression levels of top BAT-selective genes, WAT-selective genes and common fat genes in iWAT of *Hlx* transgenic mice vs. control mice. **l** Heat maps of relative expression levels of mitochondrial β-oxidation enzymes and electron transfer chain components that were significantly upregulated by at least 2-fold in the iWAT of transgenic mice. **m** Mitochondrial density (*n* = 3 mice per genotype). *Bar*, 1 µm. **n** Relative mitochondrial DNA content by qPCR (*n* = 4 mice per genotype). **o** CD31 immunofluorescence staining of iWAT of 12-week-old male mice. Shown are representative images of three mice per genotype. *Green*, CD31; *Blue*, DAPI. *Scale bar*, 200 µm. All *error bars* represent s.e.m. Two-tailed unpaired Student’s t-test was performed. *p < 0.05; **p < 0.01; ***p < 0.001

Having established the requirement of Hlx in beige adipocyte formation, we set up to examine the chronic effects of ectopic expression of Hlx on WAT browning and metabolic physiology. As Cre-mediated transgene expression can lead to irreversible recombination events during early development or at a particular time window, and *aP2-Cre* lines have been found to have recombination in endothelial cells of the heart and nonendothelial, nonmyocyte cells in the skeletal muscle^33^, we generated an *Hlx* transgenic mouse strain by directly fusing the *Hlx* cDNA downstream with the 5.4-kb *aP2* promoter. The *Hlx* transgene was selectively expressed in BAT and WAT with no expression in other tissues, including skeletal muscle and heart (Supplementary Fig. 4a). Further RT-qPCR analysis of skeletal muscle and heart with transgene-specific qPCR primers detected background signals similar to control samples. Hlx protein in the iWAT of transgenic mice was increased to about a half of its endogenous level in BAT (Fig. 5a), and was similar to the level induced by cold or by CL-316,243 agonist in the iWAT of wild-type mice (Fig. 5b). Thus, the *Hlx* transgene was expressed at a physiological protein level in the iWAT. In contrast to iWAT and eWAT, no appreciable change of Hlx protein in BAT was found (Fig. 5a and Supplementary Fig. 4b). This is consistent with our observation shown in Fig. 1 that Hlx is regulated at translational level and indicates that in the face of a robust browning of iWAT (see below), any increase of classical BAT thermogenic activity is not needed. Interestingly, similar phenomenon has also been noted in adipose tissue-specific *Ucp1* transgenic mice, where high *Ucp1* transgene expression in WAT suppresses *Ucp1* expression in BAT^34^.

On a normal chow diet, *Hlx* transgenic and littermate controls had a similar body weight (Fig. 5c and Supplementary Fig. 4c). While there was no difference in iWAT mass (Fig. 5d and Supplementary Fig. 4d), it was evident that this fat depot in the transgenic mice was much darker (Fig. 5e), and this brown-like appearance was observed in all of the transgenic mice examined compared with gender-matched littermate controls. BAT and gonadal WAT of the transgenic mice were not visually distinguishable from their counterparts of wild-type mice (Fig. 5e), except that the gonadal WAT mass was significantly lower in the transgenic mice (Fig. 5d and Supplementary Fig. 4d). *Ucp1* was induced more than 30-fold in iWAT and 3.6-fold in eWAT, and remained unchanged in BAT (Supplementary Fig. 4e). The vast majority of iWAT adipocytes of transgenic mice were multilocular (Fig. 5f) and were Ucp1-positive (Fig. 5g), which was remarkably similar to that of BAT adipocytes. These dramatic morphological and molecular changes were not seen in gonadal WAT adipocytes (Figs. 5f, g). The results indicate that a physiological increase of Hlx protein predominantly promotes browning of iWAT, and to a much less extent, the browning of gonadal fat, which together may underlie a more than 30% increase of food intake in the transgenic mice (Fig. 5h).

### Systematic remodeling of iWAT by the *Hlx* transgene

A number of genes and agents have been shown to promote browning^2, 3, 25^, however, to date, most of these studies have only examined a limited number of thermogenic genes, and an adequate molecular characterization is lacking. Indeed, whether browning is restricted to a few thermogenic genes or actually there is a systematic remodeling has been largely unclear. To better understand the process of iWAT browning in general and to grasp the extent of browning that has been achieved by the *Hlx* transgene, we performed RNA-Seq in iWAT of transgenic mice and littermate controls. Analysis of these datasets together with our previously published RNA-Seq datasets of BAT and eWAT of wild-type mice^30^ would allow us to determine whether there is a genome-wide molecular switch from WAT to BAT characteristics. We identified 1293 significantly increased genes and 1005 significantly decreased genes in the iWAT of the transgenic mice. Strikingly, 1020 of the 1293 increased genes have a significantly higher expression, and 800 of the 1005 decreased genes have a significantly lower expression, in BAT compared with eWAT of wild-type mice (Fig. 5i and Supplementary Data 1, 2). Thus, it is clear that Hlx directs a broad transcriptome extremely specific to brown/beige adipocytes. We next zoomed in to examine the expression of fat type-selective genes; these genes largely specify and maintain fat type-specific functions. Analysis of BAT and eWAT RNA-Seq data of wild-type mice produced 314 BAT-selective genes and 472 WAT-selective genes, both using a 5-fold differential gene expression cut-off. 85% of these BAT-selective genes and 56% of these WAT-selective genes were significantly induced and suppressed, respectively, in the iWAT of Hlx transgenic mice (Fig. 5j). This included all the top 26 BAT-selective genes and 7 of the top 10 WAT-selective genes we previously identified^30^, whereas most of the top 42 common fat genes remained unchanged (Fig. 5k). Remarkably, only a few fat type-selective genes displayed an opposite expression direction (Fig. 5j). Thus, Hlx robustly reprograms the whole spectrum of fat type-selective gene expression, supporting the idea that an unusually high degree of browning has indeed occurred in the transgenic mice.

Numerous genes encoding mitochondrial fatty acid β-oxidation enzymes and electron transfer chain components were upregulated at least by 2-fold in the iWAT of the transgenic mice (Fig. 5l). No induction was observed in the skeletal muscle and the heart (Supplementary Fig. 4f), consistent with their lack of expression of the transgene. Examination of mitochondrial density by transmission electron microscopy and assay of mitochondrial DNA content by qPCR clearly showed an expansion of mitochondria in iWAT (Figs. 5m, n). Increased expression of *Ucp1*, *Cidea*, and mitochondrial enzymes, and expanded mitochondrial biogenesis were also observed in iWAT adipocytes differentiated in vitro (Supplementary Fig. 4g, h), suggesting a cell-autonomous effect of the *Hlx* transgene. Functionally, the transgenic mice displayed a significantly higher oxygen consumption rate in iWAT, but not in skeletal muscle (Supplementary Fig. 4i).

Brown fat contains a dense vasculature. In light of the remarkable browning in the transgenic mice, we performed CD31 immunofluorescence staining to detect the presence of capillaries. Strikingly, the iWAT of the transgenic mice had developed an extensive capillary network (Fig. 5o), which was confirmed by direct in vivo labeling with Fluorescein labeled Lectin (Supplementary Fig. 4j). The abundant presence of capillaries was likely a secondary event elicited by increasing demands for supply of oxygen and fuels due to a WAT to brown-like fat switch. Our data shown in Fig. 5 together suggest that expression of a single transcription factor, Hlx, at a physiological level, drives a full program of iWAT browning without the requirement of any external stimuli.

### *Hlx* transgene improves glucose tolerance and prevents obesity

**Fig. 6:**
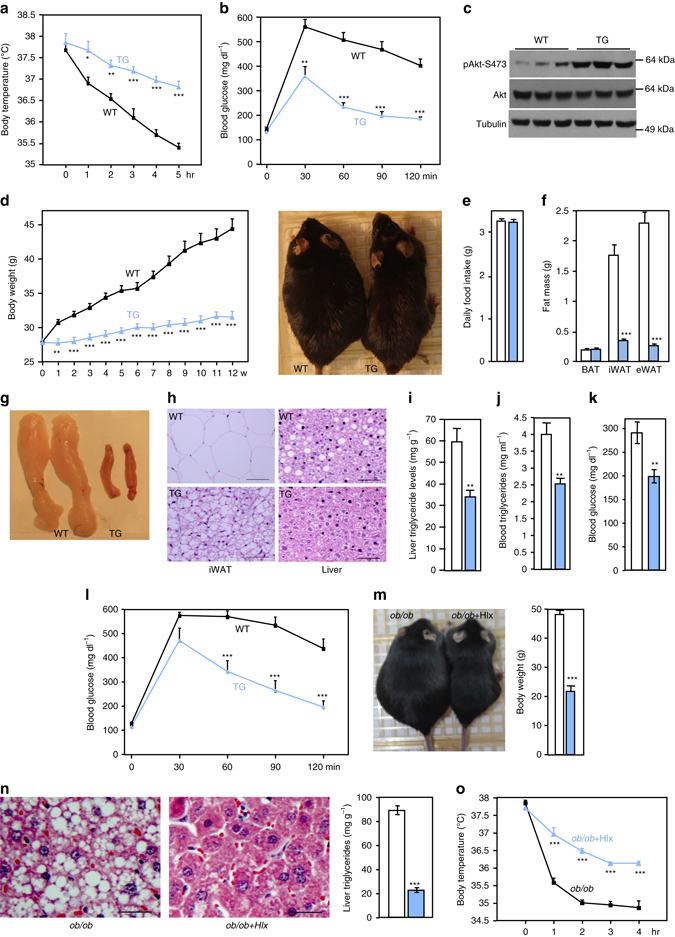
Browning of iWAT in the transgenic mice improves glucose homeostasis and prevents obesity. **a** Body temperature of 16-week-old male *Hlx* transgenic mice (*n* = 6) and littermate controls (*n* = 5) at 4 °C. **b** Glucose tolerance test of 15-month-old male *Hlx* transgenic mice (*n* = 5) and littermate controls (*n* = 7). **c** Phosphorylation level of Akt at residue S473 in iWAT. **d** Body weights of male *Hlx* transgenic mice (*n* = 7) and littermate controls (*n* = 9) on a high-fat diet for 12 weeks. **e** Daily consumption of high-fat diet of male *Hlx* transgenic (*n* = 6) and littermate control mice (*n* = 5). *White bar*, wild type; *Blue bar*, transgenic mice. **f** Fat mass of mice in (**d**). *White bar*, wild type; *Blue bar*, transgenic mice. **g** Representative image of iWAT after a 12-week high fat diet. **h** H&E staining of iWAT and liver after a 12-week highfat diet. Shown are representative images of three mice per genotype. *Scale bar*, 200 µm. **i** Hepatic triglyceride levels of mice in (**d**) after a 12-week highfat diet. *White bar*, wild type; *Blue bar*, transgenic mice. **j** Circulating triglyceride levels of mice in **d** after a 12-week high-fat diet. *White bar*, wild type; *Blue bar*, transgenic mice. **k** Steady state (5 h fasting) glucose level in a second cohort of male *Hlx* transgenic (*n* = 7) and littermate control mice (*n* = 5) after 12-week highfat diet. *White bar*, wild type; *Blue bar*, transgenic mice. **l** Glucose tolerance test of mice in (**d**) after 11-week highfat diet. Mice were fasted for 15 h. **m** Body weights of 10-week-old *ob/ob* mice (*n* = 9) and *ob/ob* mice with Hlx transgene (*n* = 6). **n** H&E staining and triglyceride content in liver of 10-week-old *ob/ob* mice (*n* = 6) and *ob/ob* mice with *Hlx* transgene (*n* = 6). *Scale bar*, 200 µm. **o** Body temperature of 10-week-old *ob/ob* mice (*n* = 5) and *ob/ob* mice with *Hlx* transgene (*n* = 4) at 4 °C. All *error bars* represent s.e.m. Two-tailed unpaired Student’s t-test was performed. *p < 0.05; **p < 0.01; ***p < 0.001

Given the extraordinary effect of Hlx on iWAT browning, we examined its phenotypic consequence. When placed at 4^o^C, the Hlx transgenic mice sustained a relatively normal body temperature and a strong induction of thermogenic gene expression (Fig. 6a and Supplementary Fig. 5a). Glucose tolerance was markedly improved in aged *Hlx* transgenic mice (Fig. 6b). Indeed, increased Akt phosphorylation was observed in iWAT (Fig. 6c), but not in the skeletal muscle, heart and, liver (Supplementary Fig. 5b). We fed the transgenic mice and littermate controls with a high-fat diet for 12 weeks. Again, at the onset of the experiments, body weights were similar between the two groups; however, they were significantly different from the first week of high-fat feeding. At the end of the experiment, while wild-type mice gained 16.5 g of body weight, the transgenic mice gained 3.7 g (Fig. 6d), which was equivalent to a body weight gain on a normal chow diet, despite similar food intake of the two groups on the high-fat diet (Fig. 6e). Similar results were obtained with female mice (Supplementary Fig. 5c). As expected, weights of iWAT and eWAT depots in the transgenic mice were much lower (Figs. 6f, g), and interestingly, iWAT still appeared brownish and maintained BAT-like morphological and molecular characteristics (Figs. 6g, h, and Supplementary Fig. 5d). Hepatic and circulating triglyceride accumulation were prevented in the transgenic mice (Figs. 6h–j). Moreover, these animals had a lower steady-state (5-hr fasting) glucose level and were more glucose tolerant (Figs. 6k, l). We also crossed the *Hlx* transgenic mice with *ob/ob* mice. As shown in Fig. 6m, n, the *Hlx* transgene reversed the obese phenotype and rescued hepatic steatosis in the *ob/ob* mice. *ob/ob* mice were highly cold sensitive; this was greatly improved by the Hlx transgene (Fig. 6o). Together, the *Hlx* transgene completely protects against both high-fat diet-induced and genetically predisposed obesity and ameliorates many of the associated metabolic abnormalities, suggesting that a WAT to brown-like fat switch can provide tremendous metabolic benefits.

### Prdm16 is a co-activator of Hlx

**Fig. 7:**
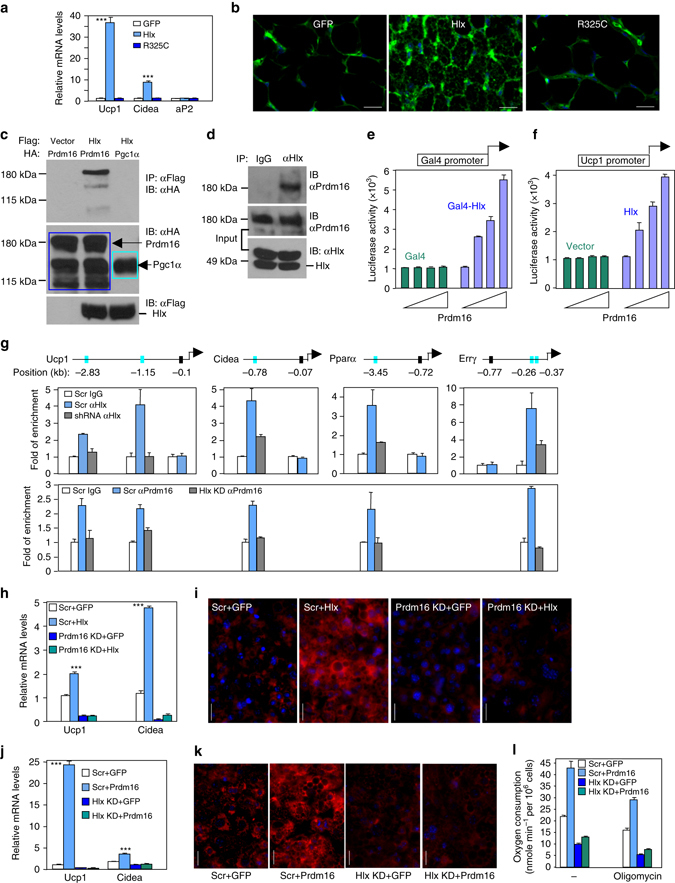
Prdm16 is a co-activator of Hlx and their thermogenic functions are inter-dependent. **a** mRNA levels of iWAT injected with overexpression adenoviruses (*n* = 4 mice per group). **b** iWAT isolated from mice in (**a**) was immunostained with a Tom20 antibody. *Green*, Tom20; *blue*, DAPI. *Scale bar*, 200 µm. **c** Flag-Hlx and HA-Prdm16 or HA-Pgc1α were transfected into HEK293 cells. Extracts were immunoblotted with an HA antibody after immunoprecipitation with a Flag antibody. **d** Cell extracts of brown adipocytes were immunoprecipitated with an antibody against Hlx and immunoblotted with an antibody against Prdm16. **e** Transcriptional activity of Gal4-Hlx fusion protein on Gal4 promoter in the presence of increased amounts of Prdm16 (*n* = 3). **f** Transcriptional activity of Hlx on the 3.1-kb Ucp1 promoter in the presence of Prdm16 (*n* = 3). **g** *Top*, *Cyan bars* depict Prdm16 peaks containing putative Hlx-binding motifs at the promoters of indicated genes, and the positions (in kb) of these peaks relative to transcription start sites are indicated. *Black bars* depict the location of control sites used in ChIP-qPCR assay. *Middle*, ChIP-qPCR analysis of Hlx association with the corresponding Prdm16 peaks in brown adipocytes with or without Hlx knockdown. Data represent average of two experiments. *Bottom*, ChIP-qPCR analysis of Prdm16 association with the corresponding Prdm16 peaks in brown adipocytes with or without Hlx knockdown. Data represent average of two experiments. **h** Brown preadipocytes were infected with Prdm16 shRNA lentivirus and differentiated. Mature adipocytes were infected with Hlx expression adenovirus, and gene expression was analyzed (*n* = 3). **i** Mature adipocytes generated as in (**h**) were stained with MitoTracker Red. *Scale bar*, 200 µm. **j** Brown preadipocytes infected with Hlx shRNA lentivirus were differentiated and infected with Prdm16 adenoviruses at day 2 and day 4. Gene expression was analyzed at day 6 (*n* = 3). **k** Mature adipocytes generated as in (**j**) were stained with MitoTracker Red. *Scale bar*, 200 µm. **l** Mature brown adipocytes generated as in (**j**) were measured for oxygen consumption without or with Oligomycin A (1 µM) (*n* = 3). All images shown are representative of three experiments. All *error bars* represent s.e.m. Two-tailed unpaired Student’s t-test was performed. *p < 0.05; **p < 0.01; ***p < 0.001

To understand the mechanistic action of Hlx, we first determined whether control of iWAT browning by Hlx depends on its DNA-binding activity. Residue Arg325 located in the homeodomain of Hlx is conserved in all homeodomain-containing transcription factors, and crystal structure and genetic studies of homeodomain proteins demonstrate that this residue is absolutely required for DNA binding^35, 36^. We mutated this Arg residue (R325C) in Hlx and generated adenoviruses. We injected Hlx mutant adenoviruses along with wild type-Hlx and GFP adenoviruses into iWAT pads. By visual inspection, iWAT tissue infected with R325C mutant or GFP adenoviruses had similar appearance, while iWAT tissue infected with wild-type Hlx was darker. As shown in Fig. 7a, b, the R325C mutant was unable to induce BAT-selective gene expression and mitochondrial biogenesis. These results clearly showed that Hlx serve as a transcription factor, not as a co-factor, to control iWAT browning.

As Hlx contains no intrinsic transcriptional activation domain, its function may depend on its interaction with transcriptional co-activators. Our data presented so far suggest a remarkable functional similarity between Hlx and Prdm16. However, Hlx does not directly regulate Prdm16 expression, as knockdown of Hlx in adipocyte culture had no effect (Fig. 2b). Although *Prdm16* mRNA expression was downregulated in iWAT of *Hlx* heterozygous mice (Fig. 4b) and upregulated in *Hlx* transgenic mice (available in RNA-Seq dataset), these are likely to be secondary effects due to impaired browning in the heterozygous mice and increased browning in the transgenic mice, respectively. Indeed, primary iWAT adipocyte culture isolated from these mice expressed similar level of *Prdm16* as primary culture isolated from their respective control mice (Supplementary Fig. 3e and Supplementary Fig. 4g). We thus considered whether Hlx utilizes Prdm16 as its co-activator. In a co-immunoprecipitation assay by overexpression in HEK293 cells, we found that Prdm16, but not Pgc-1α, was present in Hlx immunoprecipitated complex (Fig. 7c). This interaction between Hlx and Prdm16 could be recapitulated in brown adipocytes with their endogenous protein levels (Fig. 7d). We next examined whether Prdm16 co-activates Hlx in a luciferase reporter assay. We fused Hlx with the Gal4 DNA-binding domain, and this fusion protein alone had no effect on Gal4 promoter-directed luciferase reporter. Prdm16 dose-dependently increased the transcriptional activity of Gal4-Hlx fusion protein, but did not increase the activity of Gal4 DNA-binding domain itself (Fig. 7e). We also tested a 3.1-kb *Ucp1* promoter that contains Hlx binding sites (see below). Again, Prdm16 dose-dependently increased *Ucp1* promoter activity in the presence of Hlx (Fig. 7f), and Pgc-1α had no effect (Supplementary Fig. 6a, b). Thus, Prdm16 is capable of co-activating Hlx. As expected, Prdm16 interacted with the DNA-binding defective Hlx mutant (R325C) (Supplementary Fig. 6c), but was unable to co-activate it on the *Ucp1* promoter (Supplementary Fig. 6d).

The above data led to the idea that Hlx recruits Prdm16 to the promoters of some Hlx-regulated genes, and as such, it is expected that binding sites of Hlx and Prdm16 at these gene promoters may overlap. To this end, we analyzed the published Prdm16 ChIP-Seq dataset of brown adipocytes^37^ to search for presence of putative Hlx-binding motif within Prdm16 peaks, with a focus on promoter regions (<10 kb upstream of transcription start sites). Among the 7 Hlx-regulated genes described in Fig. 2b, *Ucp1, Cidea, Pparα* and *Errγ* were found to contain Hlx-binding motif sequences located within Prdm16 peaks at their promoters, as depicted in Fig. 7g, top panel (and Supplementary Fig. 6e). ChIP-qPCR analysis in brown adipocytes showed that Hlx associated with these Hlx-binding motifs but not with control sites (Fig. 7g and Supplementary Fig. 6f). Importantly, in Hlx knockdown brown adipocytes, both Hlx and Prdm16 association with these motifs were diminished (Fig. 7g). Thus, Hlx indeed recruits Prdm16 to these bona fide Hlx-binding sites. We then tested the functional dependency between Hlx and Prdm16. Adenoviral overexpression of Hlx stimulated *Ucp1* and *Cidea* expression and mitochondrial biogenesis in brown adipocytes, but not in Prdm16 knockdown adipocytes (Figs. 7h, i). Similarly, knockdown of Hlx largely abolished Prdm16-induced *Ucp1* and *Cidea* expression and mitochondrial biogenesis as well as oxygen consumption (Figs. 7j–l). Collectively, our data suggest that Prdm16 is a co-activator of Hlx and their thermogenic functions are highly inter-dependent.

## Discussion

We report here that Hlx, at least in part through Prdm16-mediated co-activation, directly drives a full program of thermogenesis and a robust WAT to brown-like fat switch, which in turn improves glucose homeostasis and prevents both genetic and high-fat diet-induced obesity and fatty liver. Results from fat-specific injection of Hlx knockdown and overexpression viruses not only confirm that the iWAT remodeling phenotypes observed in the *Hlx* knockout and transgenic mice are fat tissue-autonomous effects, but also suggest that iWAT remodeling can be accomplished very rapidly in response to changes of Hlx level. These complementary studies reveal Hlx as a major transcription factor governing iWAT browning. Our work also has important implications for cellular reprograming. Reprograming of human pluripotent stem cells or other cell lineages into brown adipocytes is a promising avenue to model and treat metabolic diseases^38, 39^. Our results suggest that Hlx may be considered as a vital choice when constructing a powerful toolkit for cellular reprograming into brown adipocytes.

Expression of thermogenic transcriptional regulators such as Pgc-1α was known to be modulated by β3-adrenergic signaling at transcriptional level. One unexpected finding is that Hlx is translationally upregulated by β3-adrenergic activation through suppression of the translational inhibitor 4E-BP1. Interestingly, *4E-BP1* knockout mice display browning of iWAT^32^. Our results reveal a novel regulatory pathway utilized by β3-adrenergic signaling to control the expression of its downstream thermogenic regulators, and provide a potential mechanism underlying the browning phenotype of the *4E-BP1* knockout mice. Another distinct feature of Hlx is that, unlike transcription factors, such as Pparγ, Cebpβ, and Ebf2, which control both brown adipogenesis and BAT-selective gene expression^39, 40^, Hlx has no effect on adipogenesis per se. Instead, Hlx exclusively regulates BAT-selective gene expression and mitochondrial biogenesis. Our observations suggest that Hlx is a dedicated transcription factor uniquely equipped to control WAT browning. It is important to note that our studies presented here investigated the browning effects of Hlx on differentiating and/or mature adipocytes, a process often referred to as transdifferentiation. Whether Hlx plays a role in adipocyte progenitors to establish beige preadipocyte lineage is unknown.

iWAT of heterozygous *Hlx* mice has a significant defect in beige adipocyte formation. This defect is attributed to haploinsufficiency in iWAT, not in other tissues, as supported by our in vitro primary cell culture studies and in vivo fat-specific virus injection studies. The inability to maintain body temperature during cold suggest that heat production by beige adipocytes is indeed important for thermoregulation despite their low Ucp1 levels compared with classical BAT. Moreover, these mice are glucose intolerant during aging, and although having a similar body weight as control mice on a high-fat diet, they develop hepatic steatosis. Interestingly, deletion of *Prdm16* by *adiponectin-Cre* leads to a similar phenotype, that is, selective defect in iWAT browning, high-fat diet-induced, body weight-independent hepatic steatosis, and glucose intolerance^28^. While reduced energy expenditure due to loss of beige adipocytes might be responsible, alternative mechanisms are equally possible. In this regard, it is important to note that a recent study demonstrated that Nrg4, a BAT-enriched secreted factor that is also expressed in WAT, protects against high-fat diet-induced hepatic steatosis^41^. Thus, whether loss of beige adipocytes alters the secretome, which in turn alters glucose and lipid metabolism in fat and other tissues, is warranted for future investigation. Nevertheless, our results indicate that a normal number of beige adipocytes present in wild-type mice might be indispensable for cold tolerance and glucose and lipid homeostasis. The importance of these “endogenous” beige adipocytes in physiology has been unclear until recently^28^.

When expressed at a physiological protein level similar to that of induced by β3 agonist or cold exposure, Hlx alone promotes a nearly complete remodeling of iWAT. The extensive vascularization is particularly interesting, but how exactly this occurs is unclear. On the other hand, this expanded capillary network could in turn further boost the browning effect of the *Hlx* transgene, an idea that is supported by studies showing a functional relationship between beige adipocyte formation and vasculature^42–44^. Interestingly, whereas cross-communication between BAT and WAT has been observed in other animal models^34, 45, 46^, the Hlx transgenic mice do not show a feedback suppression of classical BAT function. Instead, browning of iWAT in the transgenic mice requires a 30% more food consumption to maintain energy homeostasis and normal body weight, indicating that these brown-like adipocytes are active in thermogenesis at basal conditions. The functional effects of Hlx-induced browning are more striking when the transgenic mice were challenged by cold, aging, fed a high fat diet, or crossed with *ob/ob* mice. In particular, the Hlx transgene significantly improves glucose tolerance during aging and completely protects against both high-fat diet-induced and genetic-predisposed obesity. Such a dramatic phenotype is most likely attributed to the systematic remodeling of iWAT that includes increased mitochondrial and vascular densities; a mere upregulation of a few thermogenic effector genes is certainly important, but unlikely to be sufficient.

Hlx is both necessary and sufficient for browning of iWAT, similar to Prdm16^26–28^. This, along with the exclusive dependency of Hlx on its DNA-binding activity, raises the possibility that Hlx is a transcriptional partner of Prdm16. Our data showing their molecular and genetic interactions and functional inter-dependency strongly suggest that Hlx is a major mediator of Prdm16. Future work will aim to further dissect Hlx-associated transcriptional machinery, and understand in detail how environmental cues and pathological conditions impact Hlx translation and/or activity. In summary, Hlx drives a full program of thermogenesis and serves as a powerful regulator for systematic and robust browning of WAT. As conversion of WAT or other cell types to brown-like fat has been considered as a therapeutic strategy for obesity and metabolic diseases, Hlx may represent a potential molecular target.

## Methods

### Adipocyte differentiation and treatment

Immortalized BAT preadipocyte cell line was generated previously^47^. Primary iWAT preadipocytes were isolated from 2-week-old C57BL6/J mice through collagenase digestion. Differentiation was performed. Briefly, at day −2, brown preadipocytes at 70% confluent were changed to medium containing 20 nM insulin and 1 nM 3,3′,5-triiodo-L-thyronine (differentiation medium). At day 0, differentiation was induced by culturing cells in differentiation medium supplemented with 0.5 mM isobutylmethylxanthine, 0.5 µM dexamethasone, and 0.125 mM indomethacin for 48 h. After this induction period, cells were changed back to differentiation medium. iWAT adipogenesis was similarly performed by culturing confluent cells in medium containing 850 nM insulin, 1 nM 3,3′,5-triiodo-L-thyronine, 0.5 mM isobutylmethylxanthine, 0.5 µM dexamethasone, and 0.125 mM indomethacin. At day 6, both brown and iWAT adipocytes were fully differentiated and harvested. In some experiments, differentiated adipocytes were treated with Forskolin (10 µM) (Acros Organics, Cat#BP2520) or CL-316243 (10 µM) (Tocris, Cat#1499) as indicated.

Human preadipocyte SGBS cells were differentiated following an established protocol^48^. Briefly, SGBS preadipocytes were cultured in DMEM/F12 medium (Invitrogen, Cat#31330-038) containing 10% fetal calf serum, biotin (0.33 μM, Sigma, Cat#B-4639) and panthotenat (0.17 μM, Sigma, Cat#P-5155). At day 0, cell differentiation was started in DMEM/F12 medium containing 0.33 μM biotin, 0.17 μM panthotenat, 2 μM rosiglitazone (Cayman, Cat#71740), 25 nM dexamethasone (Sigma, Cat#D-1756), 0.5 mM methylisobuthylxantine (Sigma, Cat#I-5879), 0.1 μM cortisol (Sigma,Cat#H-0888), 0.01 mg ml^−1^ transferring (Sigma, Cat#T-2252), 0.2 nM triiodotyronin (Sigma, Cat#T-6397), and 20 nM human insulin (Sigma,Cat#I-1507). At day 5, cells were cultured in DMEM/F12 containing 0.1 μM cortisol, 0.01 mg ml^−1^ transferrin, 0.2 nM triiodotyronin, and 20 nM human insulin. The medium was changed every 4 days and cells were fully differentiated at day 14.

### Plasmids and viruses

Full-length *Hlx* (mouse and human) and *Prdm16* cDNA (mouse) were generated by PCR and verified by sequencing. Adenoviral *Hlx* and *Prdm16* overexpression plasmid was constructed using AdEasy-1 system, and viruses were produced and purified with cesium chloride ultracentrifugation^49^. *Hlx* and *Prdm16* shRNA knockdown lentiviral constructs were generated using psp-108 vector (Addgene), and viruses were produced by co-transfection along with plasmids pMD2.G (Addgene) and psPAX2 (Addgene) into HEK293T cells. All viral titers were predetermined, and same number of viral particles was used for experimental and control samples. Differentiating adipocytes were infected with adenoviruses at 100% infection efficiency. Preadipocytes were infected with lentiviruses, selected with puromycin, and re-plated for differentiation. *Hlx* knockdown targeting sequences are: shRNA-1, 5′GGCGCAGAAGGACAAGGACAAGGAAGCGG3′; shRNA-2, 5′GCATCAAACCACGGTCATCAA3′. *Prdm16* knockdown targeting sequences are: 5′GAAGAGCGTGAGTACAAAT3′. *4E-BP1* knockdown targeting sequences are: shRNA-1, 5′ CAGGATTATCTATGACCGGAA 3′; shRNA-2, 5′ CCAAAGGACCTGCCAGCCATT 3′.

### Animal studies

All animal studies were performed according to procedures approved by The University of Massachusetts Medical School’s Institutional Animal Care and Use Committee. *Hlx* heterozygous knockout mice (Stock# 008315) and heterozygous *leptin*-deficient mice (Stock# 000632) were purchased from Jackson Laboratory. Full-length *Hlx* cDNA was fused downstream with the 5.4-kb *aP2* (*Fabp4*) promoter. The transgenic DNA fragment was gel-purified and injected into fertilized embryos harvested from C57BL/6 × SJL hybrid mice. Transgenic lines were backcrossed with C57BL/6 for at least three generations. Normal diet containing 4% (w/w) fat and high-fat diet containing 36% (w/w) fat were purchased from Harlan Teklad and Bioserv (Product# F3282), respectively. Food consumption was measured daily for individually caged mice. β3-adrenergic agonist CL-316243 (Tocris, Cat#1499) was intraperitoneally injected into mice at 0.5 mg kg^−1^ body weight. For glucose tolerance test, glucose was intraperitoneally injected into mice at 2 g kg^−1^ body weight after 16-hr fasting. For acute cold exposure, mice were individually caged and placed in a 4 °C cold room, and core body temperature was measured with the Microtherma 2 rectal probe (Thermoworks). Gender-matched littermate controls were used in all the experiments of *Hlx* knockout and transgenic mouse studies.

### In vivo lentivirus and adenovirus injection into iWAT

High-titer Hlx knockdown lentiviruses (1 × 10^9^ transducing unit ml^−1^) for in vivo injection were prepared^50^. Two days after co-transfection into HEK293T cells with psp-108 vector and helper plasmids pMD2.G and psPAX2, the virus-containing medium was collected, pre-cleared with 3000 g centrifugation for 5 min, and then passed through a 0.45 μm filter (Millipore). Subsequently, the virus-containing medium was overlaid on a 10% sucrose-containing buffer (50 mM Tris-Hcl, pH = 7.4, 100 mM NaCl, 0.5 mM EDTA) at a 4:1 v/v ratio, and centrifuged at 10,000 g for 4 h at 4 °C. After centrifugation, the supernatant was carefully removed, and the viruses were resuspended with PBS and placed at 4 °C for recovery overnight. Adenoviruses were purified with cesium chloride ultracentrifugation. 3-month-old male wild-type C56BL6 mice were used for viral injection. Experimental viruses and control viruses were injected into the left iWAT pad and right iWAT pad of the same animal, respectively. For Hlx knockdown, lentiviruses were injected into five spots at 1 × 10^8^ transducing unit per injection to cover the whole fat pad. For Hlx overexpression, adenoviruses were injected into the center of iWAT pads within a marked area of approximate 0.5 cm in diameter at 1 × 10^10^ pfu per injection twice. At day 8, the mice were sacrificed, and whole iWAT fat pads (knockdown) or iWAT tissues from the injected area (overexpression) were collected.

### Histology and immunofluorescence

For H&E staining, tissues were fixed with 10% formalin and paraffin-embedded, and then standard procedures were performed. To label blood vessels in vivo, Fluorescein labeled Griffonia Simplicifolia Lectin I (4 µg per mouse, Vector Laboratories, Cat# FL-1101) was gently injected into 2-month-old male mice via tail vein. After 20 min, the mice were sacrificed and iWAT tissues were isolated and carefully minced with scissors. The iWAT tissues were washed twice with pre-warmed PBS and cover-slipped by Fluoromount-G (SouthernBiotech, Cat#0100-01). For immunofluorescence staining, formalin-fixed and paraffin-embedded tissue sections were deparaffinized and rehydrated through graded ethanol solutions. After pre-incubation with a blocking buffer (PBS containing 5% normal goat serum and 0.3% Triton X-100) for 60 min, slides were incubated with Ucp1 antibody (Sigma, Cat#U6382) (1:500 dilution), Tom20 antibody (Santa Cruz, Cat#sc-17764) (1:100 dilution), or CD31 antibody (1:250) (Millipore Cat#MAB2148-C) in blocking buffer at 4 °C overnight. Subsequently, the slides were washed, and incubated with Alexa Flour 488-conjugated or 594-conjugated secondary antibody and DAPI for 60 min. Images were acquired and processed with the same setting for transgenic mice or knockout mice and control littermates.

### Transmission electron microscopy

Transmission electron microscopy were performed as described^47^. Samples were fixed in 2.5% glutaraldehyde in PBS (pH 7.2), washed, and then post-fixed in 1% osmium tetroxide. The fixed samples were dehydrated through a graded series of ethanol to 100%, followed by two changes of propylene oxide and finally into a 50:50 (v/v) mixture of propylene oxide: epoxy resin (SPON 812/Araldite 502), and left overnight to infiltrate. Samples were processed through two changes of fresh epoxy resin and embedded, allowing the blocks to polymerize 48 h at 70°C. Ultrathin sections were cut on a Reichart-Jung ultramicrotome using a diamond knife. The sections (64 nm thick) were collected and mounted on copper support grids and contrasted with lead citrate and uranyl acetate, and examined on a Philips CM 10 transmission electron microscope at 80 kV accelerating voltage. We used ImageJ software to calculate mitochondrial area and cellular area in individual micrographs, and mitochondrial density was expressed as a percentage of cellular area.

### MitoTracker staining

Adipocytes were stained with MitoTracker Red CMXRos (250 nM) (Molecular Probes, Cat# M7512) in DMEM containing 10% FBS at 37 °C for 30 min. Cells were then gently washed twice with DMEM containing 10% FBS, followed by fixation with 4% formaldehyde at 37 °C for 15 min. Cells were gently rinsed twice with PBS, permeabilized with ice-cold 100% methanol at −20 °C for 10 min, and washed twice with PBS. Finally, the cells were incubated with DAPI for 60 min at room temperature.

### Polysome analysis

This was performed as described^51^. Briefly, fully differentiated brown adipocytes on a 15-cm plate were treated with or without Forskolin or CL-316243 for 9 h. The cells were treated with 100 µg ml^−1^ cycloheximide (Sigma, Cat#C7698) for 5 min, harvested in 5 ml ice-cold PBS containing 100 µg ml^−1^ cycloheximide, and centrifuged at 4 °C. 425 µl of hypotonic buffer (5 mM Tirs-HCl, pH 7.5; 2.5 mM MgCl_2_; 1.5 mM KCl and protease inhibitor cocktail, EDTA-free), 5 µl of 10 mg ml^−1^ cycloheximide, 1 µl of 1 M DTT and 100 units of RNasin (Promega, Cat#N251A) were added into cell pellet and vortexed for 5 s, and then 25 µl of 10% TritonX-100 and 25 µl of 10% sodium deoxycholate were added and vortexed for 5 s. Samples were centrifuged at 4 °C, and OD_260_ of supernatants was measured using NanoDrop. 18 OD_260_ units were loaded onto sucrose gradient (10–50%) and centrifuged at 35,000 rpm with a SW40 rotor for 2 h at 4 °C. Each sucrose gradient fraction was collected and RNA was isolated for RT-qPCR analysis. Levels of mRNA in polysome fractions were normalized with the sum of mRNA levels in all the fractions.

### RNA-Seq and data analysis

Total RNA was isolated from iWAT of 12-week-old male *Hlx* transgenic and littermate control mice (*n* = 3 mice per genotype) using TRIzol Reagent. 4 µg of total RNA was used to construct biologically triplicate sequencing libraries essentially according to Illumina TruSeq RNA Sample Preparation v2 Guide. Amplified libraries were sequenced on an Illumina HiSeq 2000 system.

RNA-Seq 100 base pair single-end reads were binned according to six base pair barcode using a perl script written in house. Reads with barcode removed were mapped to the mouse genome (mm10) using TopHat, followed by running Cufflinks to assemble and quantify transcriptome^52^. Transcript abundances were presented as FPKM (Fragments Per Kilobase of transcript per Million mapped reads). Differential expression analysis was performed using Cuffdiff^52^. Genes with q value <0.05 and averaged FPKM value >1 in at least one genotype were defined as Hlx-regulated genes.

We used previously published BAT and eWAT RNA-Seq dataset (GEO accession number GSE56367)^30^ to identify fat type-selective genes with a 5-fold differential expression cut-off and FPKM value >5 in at least one tissue.

### Search of putative Hlx-binding motif

The PWM file of Hlx primary motif (generated by seed-and-wobble algorithm) was downloaded from UniProbe database (http://thebrain.bwh.harvard.edu/uniprobe)^53, 54^. The motif was trimmed by information content no less than 0.4 in both ends by R/Bioconductor package motifStack (version 1.14.0). Published Prdm16 peaks of brown adipocytes^37^ were downloaded and annotated by R/Bioconductor package ChIPpeakAnno (version 3.5.1)^55^. The sequences of the Prdm16 peaks were used for motif searching by the match PWM function in R/Bioconductor package Biostrings (version 2.38.2). The minimum score for matching is 80%.

### ChIP-qPCR

Standard ChIP assays were performed^30^ with antibodies against Hlx (Millipore, Cat# 09-084), Prdm16 (R&D Systems, Cat# AF6295), or normal rabbit IgG. Cells (1 × 10^6^) were fixed in 1% formaldehyde for 5 min at room temperature and then washed with cold PBS containing protease inhibitor cocktail (Roche) and pelleted by centrifugation. The cell pellets were suspended in 100 µl ChIP lysis buffer (50 mM Tris-HCl pH 8.1, 10 mM EDTA, 1% SDS, and protease inhibitors). Fragmentation of the DNA (100–200 bp) was achieved by a 3 min sonication (10 s pulse on and 5 s pulse off) on ice with ultrasonic processor (Cole Parmer) at 40% amplitude. The sheared chromatin was cleared by centrifugation at 16,000 g for 10 min at 4 °C and diluted 10-fold in ChIP IP buffer (16.7 mM Tris-HCl pH 8.1, 1.2 mM EDTA, 1.1% Triton X-100, 0.01% SDS, and 167 mM NaCl). 50% protein A-sepharose slurry (GE Healthcare #17-0780-01) was added to the sample and incubated for 1 h at 4 °C with rotation. The precleared chromatin solution was then incubated with 3 µg indicated antibodies overnight. The antibodies were captured with 30 µl of 50% protein A-sepharose slurry for 1 h, and the beads were then washed five times. Crosslinking was reversed by incubation at 65 °C overnight and DNA fragments were purified with QIAquick PCR Purification Kit. Immunoprecipitate signal was normalized with input signal; both were measured by real-time qPCR.

### Luciferase reporter assays

Cos-7 cells in 48-well plates were transfected with luciferase reporters, CMV-β-galactosidase plasmids and indicated expression constructs, using lipofectamine 2000 (Invitrogen). Vector plasmids were used to adjust the total amount of plasmids per well. Luciferase activity was measured 48 h after transfection.

### Western blot analysis and co-immunoprecipitation

For western blot analysis, cultured adipocytes or adipose tissue samples with equal amounts were homogenized in lysis buffer [100 mM NaCl, 50 mM Tris (pH7.5), 0.5% Triton X-100, 5% (w/v) glycerol]. Lysates were cleared at 12,000 g for 10 min. Supernatants were quantified for protein content, separated by SDS-PAGE, and immunoblotted with antibodies to Hlx (Millipore, Cat# 09-084), Ucp1 (Sigma, Cat# U6382), Phospho-Akt (Ser473) (Cell Signaling Technology, Cat #4060), total Akt (Cell Signaling Technology, Cat #4691), or Tubulin (DSHB#E7-S).

To examine the interaction between Hlx and Prdm16 in HEK293 cells, Flag-Hlx or vector and HA-Prdm16 were transfected into HEK293 cells. After 48 h, cell extracts were generated in the above lysis buffer, and incubated for 3 h with anti-Flag M2 affinity gel (Sigma, A2220). The beads were washed four times with washing buffer [100 mM NaCl, 50 mM Tris (pH 7.5), 0.1% NP-40, 3% glycerol]. Immunoprecipitates were analyzed by Western blotting.

To examine the endogenous interaction between Hlx and Prdm16, differentiated brown adipocytes were lysed in the above lysis buffer. Hlx antibody or IgG was added into the cleared cell extracts, and incubated for overnight at 4 °C. Protein-A beads were then added and incubated for 3 h at 4 °C. Beads were washed five times with above washing buffer. Immunoprecipitates were analyzed by Western blotting with a Prdm16 antibody (R&D Systems, Cat# AF6295).

Original, uncropped blots can be found in Supplementary Fig. 7.

### Gene expression

Total RNA was extracted using TRIzol, and an equal amount of RNA was reverse transcribed. Gene expression was measured by quantitative real-time PCR (qPCR) using SYBR Green, and normalized to ribosomal 36B4. Primer sequences are available upon request.

### Statistical analysis

The sample size was chosen based on our experience and similar work in the literature. No randomization was used, and the investigators who performed mouse experiments were not blinded to genotypes. No animals were excluded from data analysis. Two-tailed unpaired Student’s t-test was used for statistical analysis unless otherwise indicated. N represents biological replicates. *p* < 0.05 was considered significant. Data are presented as mean ± s.e.m.

### Data availability

RNA-Seq data generated in the current study have been deposited in Gene Expression Omnibus under the accession code GSE78143. The authors declare that all other relevant data supporting the findings of this study are available within the paper and its Supplementary Information files, or from the corresponding authors upon request.

## Electronic supplementary material

Supplementary Information

Supplementary Data 1

Supplementary Data 2
