# Supplementary material for: Transcription factor Hlx controls a systematic switch from white to brown fat through Prdm16-mediated co-activation
Source: Nat Commun. 2017 Jul 12;8:68. doi: 10.1038/s41467-017-00098-2 (PMC5507986; doi:10.1038/s41467-017-00098-2)
Supplement: Supplementary file 1 — Supplementary Information [file 41467_2017_98_MOESM1_ESM.pdf]

File Name: Supplementary Information
Description: Supplementary Figures.

File Name: Supplementary Data 1
Description: Upregulated genes in the iWAT of Hlx transgenic mice and their expression levels in BAT and eWAT of wild type mice.

File Name: Supplementary Data 2
Description: Downregulated genes in the iWAT of Hlx transgenic mice and their expression levels in BAT and eWAT of wild type mice.

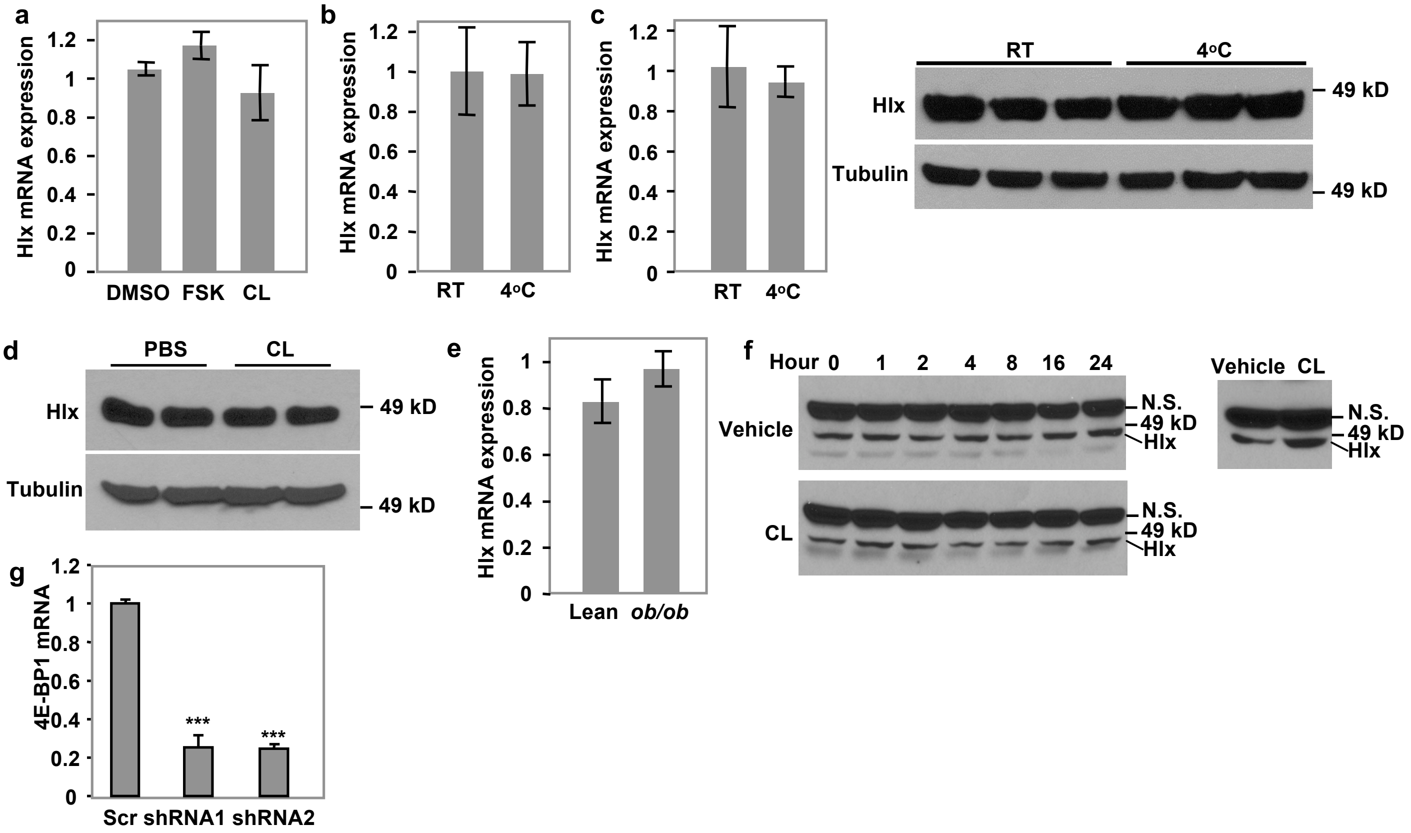

Supplementary Figure 1. Hlx expression in adipocyte.

(a) Hlx mRNA expression in brown adipocytes treated with Forskolin or CL-316,243 for 12 hr (n=5). **(b)** Hlx mRNA expression in iWAT of 12-week-old male mice at room temperature or cold challenged for 6 hr (n=3). **(c)** Hlx mRNA and protein in BAT of 12-week-old male mice at room temperature or cold challenged for 6 hr (n=3). **(d)** Western blot analysis of Hlx protein in BAT of 12-week-old male mice. Mice were intraperitoneally injected with a single dose of CL-316,243 or PBS, and were sacrificed 24 hr later. **(e)** Hlx mRNA expression in iWAT of 3-month-old *ob/ob* mice and C57BL6 mice (n=3). **(f)** In vitro differentiated brown adipocytes were treated with CL-316,243 for 18 hr, followed with protein synthesis inhibitor cycloheximide. Samples were collected at different time points to examine Hlx protein. Right panel shows Hlx protein levels of cycloheximide treated and untreated samples at 0 time point on the same gel. N.S. indicates a non-specific band. **(g)** 4E-BP1 mRNA expression in brown adipocytes expressing lentiviral 4E-BP1 knockdown constructs (n=3).

All error bars represent s.e.m. Two-tailed unpaired Student's t-test was performed. ***$p<0.001$.

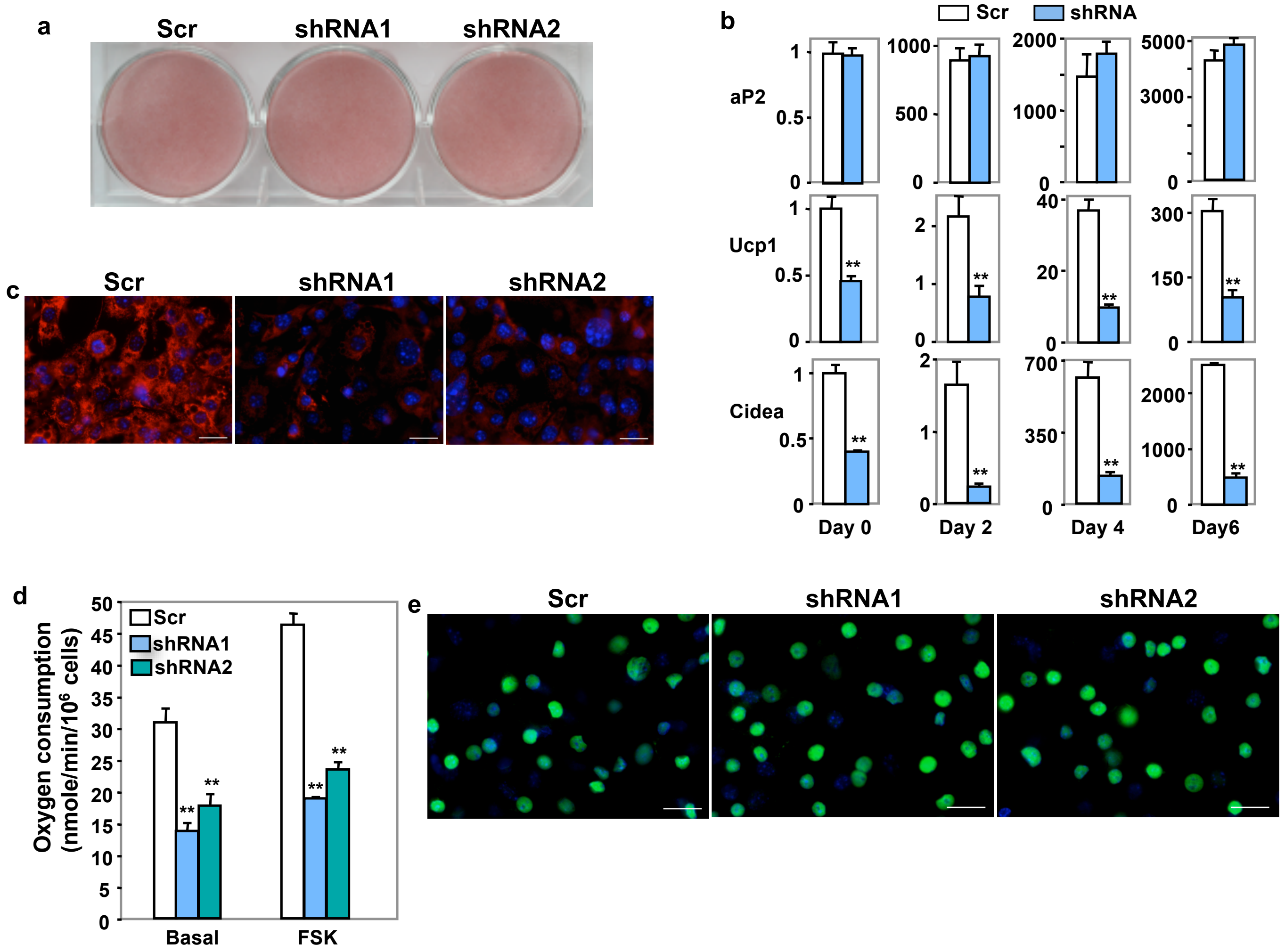

Supplementary Figure 2. Hlx regulates thermogenesis, but not general differentiation.

(a) Immortalized brown preadipocytes were infected with Hlx knockdown lentiviruses and differentiated into mature adipocytes. Oil Red O staining to visualize triglycerides. **(b)** Gene expression (n=3) was analyzed at indicated time points of adipogenesis and normalized to levels at Day 0. **(c)** MitoTracker Red staining to visualize mitochondria in mature brown adipocytes generated as in (**a**). Scale bar, 200 μm. **(d)** Mature brown adipocytes generated as in (**a**) were either untreated or treated with Forskolin for 12 hr. Oxygen consumption was measured (n=3) with a Clark-type electrode in the presence of FCCP (1 μM). **(e)** Primary iWAT preadipocytes were infected with Hlx knockodown lentiviruses, differentiated into mature adipocytes, and then immunostained with a C/ebpα antibody.Green, C/ebpα; Blue, DAPI. Scale Bar, 200 μm.

All error bars represent s.e.m. Two-tailed unpaired Student's t-test was performed. $^{**}p<0.01$.

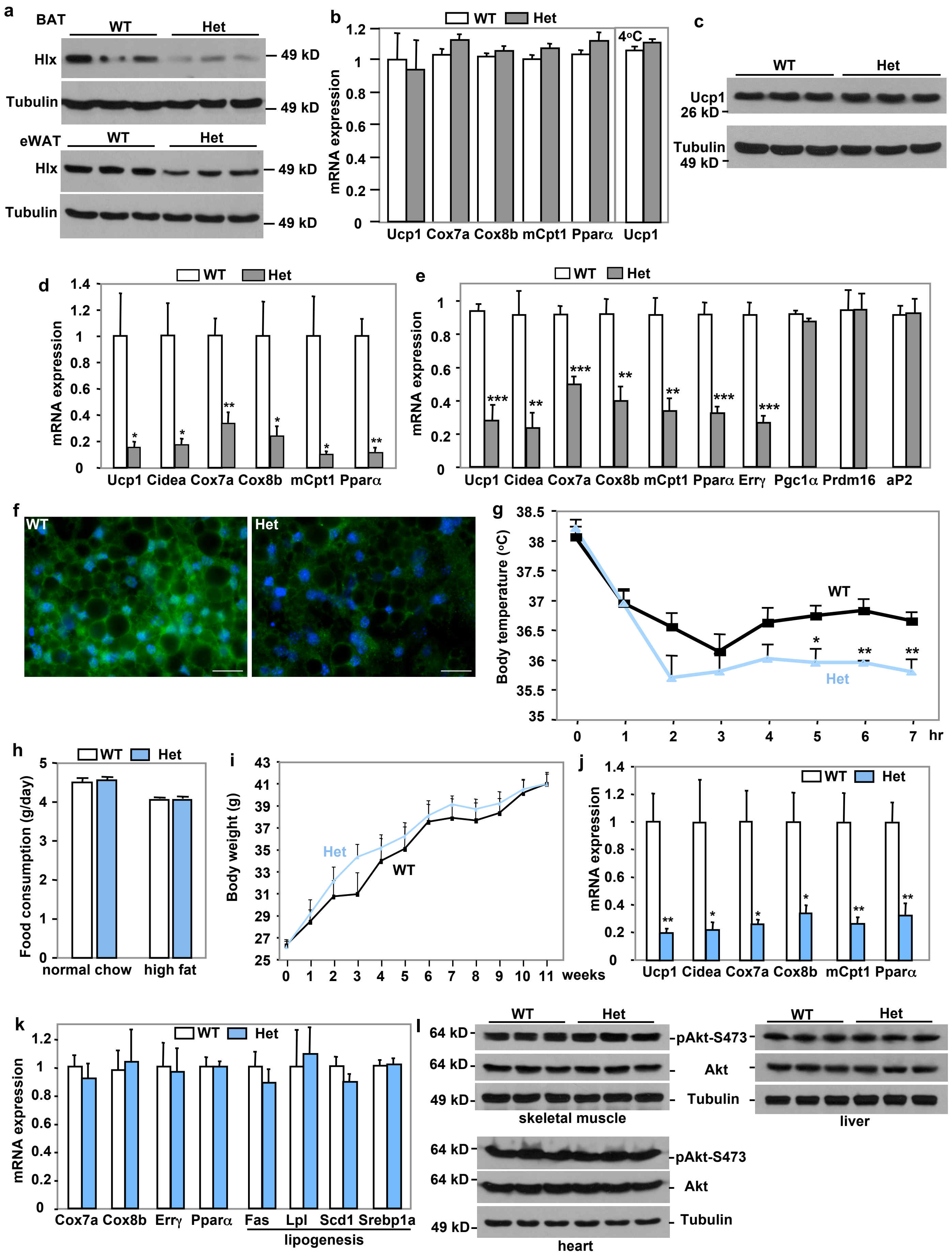

a
BAT
WT
Het
Hlx
— 49 kD
Tubulin
— 49 kD
eWAT
WT
Het
Hlx
— 49 kD
Tubulin
— 49 kD
b
WT
Het
mRNA expression
1.2
1
0.8
0.6
0.4
0.2
0
4°C
Ucp1
Cox7a
Cox8b
mCpt1
Pparα
Ucp1
c
WT
Het
Ucp1
26 kD
Tubulin
49 kD
d
WT
Het
mRNA expression
1.4
1.2
1
0.8
0.6
0.4
0.2
0
*
*
**
*
*
**
Ucp1
Cidea
Cox7a
Cox8b
mCpt1
Pparα
e
WT
Het
mRNA expression
1
0.8
0.6
0.4
0.2
0
***
**
***
**
**
***
***
Ucp1
Cidea
Cox7a
Cox8b
mCpt1
Pparα
Errγ
Pgc1α
Prdm16
aP2
f
WT
Het
g
Body temperature (°C)
38.5
38
37.5
37
36.5
36
35.5
35
WT
Het
*
**
**
0
1
2
3
4
5
6
7
hr
h
WT
Het
Food consumption (g/day)
5
4
3
2
1
0
normal chow
high fat
i
Body weight (g)
43
41
39
37
35
33
31
29
27
25
Het
WT
0
1
2
3
4
5
6
7
8
9
10
11
weeks
j
WT
Het
mRNA expression
1.4
1.2
1
0.8
0.6
0.4
0.2
0
**
*
*
*
**
**
Ucp1
Cidea
Cox7a
Cox8b
mCpt1
Pparα
k
WT
Het
mRNA expression
1.2
1
0.8
0.6
0.4
0.2
0
Cox7a
Cox8b
Errγ
Pparα
Fas
Lpl
Scd1
Srebp1a
lipogenesis
l
WT
Het
64 kD
pAkt-S473
64 kD
Akt
49 kD
Tubulin
skeletal muscle
WT
Het
liver
64 kD
pAkt-S473
64 kD
Akt
49 kD
Tubulin
heart

Supplementary Figure 3. Hlx heterozygous knockout mice are defective in iWAT browning.

(a) Hlx protein levels in BAT and eWAT of 3-month-old mice. **(b)** mRNA expression in BAT of 3-month-old mice at 23°C or 4°C for 6 hr. n=4 mice $group^{-1}$. **(c)** Ucp1 protein in BAT of 3-month-old mice.

(d) 16-week-old Hlx mice (n=3 mice $group^{-1}$) were placed at 4°C for 6 hr. Gene expression in iWAT was analyzed. **(e)** Primary iWAT preadipocyts were isolated (n=4 mice $group^{-1}$) and differentiated in vitro. mRNA expression was analyzed. **(f)** Primary adipocytes generated in **(d)** were immunostained with a Tom20 antibody. Shown are representative images of three mice per genotype. Green, Tom20; blue, DAPI.

Scale bar, 200 µm. **(g)** Body temperature of 3-month-old mice (n=5-7 mice $group^{-1}$) at 4°C. **(h)** Daily food consumption of 3-month-old mice (n=6 mice $group^{-1}$). **(i)** Body weights of male mice (n=5-6 mice $group^{-1}$) on a high fat diet for 11 weeks. **(j)** Gene expression in iWAT of mice in (**i**) after 11 week high fat diet.

(k) Hepatic gene expression in 3-month-old male mice (n=5 mice $group^{-1}$). **(l)** Phosphorylation level of Akt at residue S473 in skeletal muscle, liver and heart.

All error bars represent s.e.m. Two-tailed unpaired Student's t-test was performed. *$p<0.05$; **$p<0.01$; ***$p<0.001$.

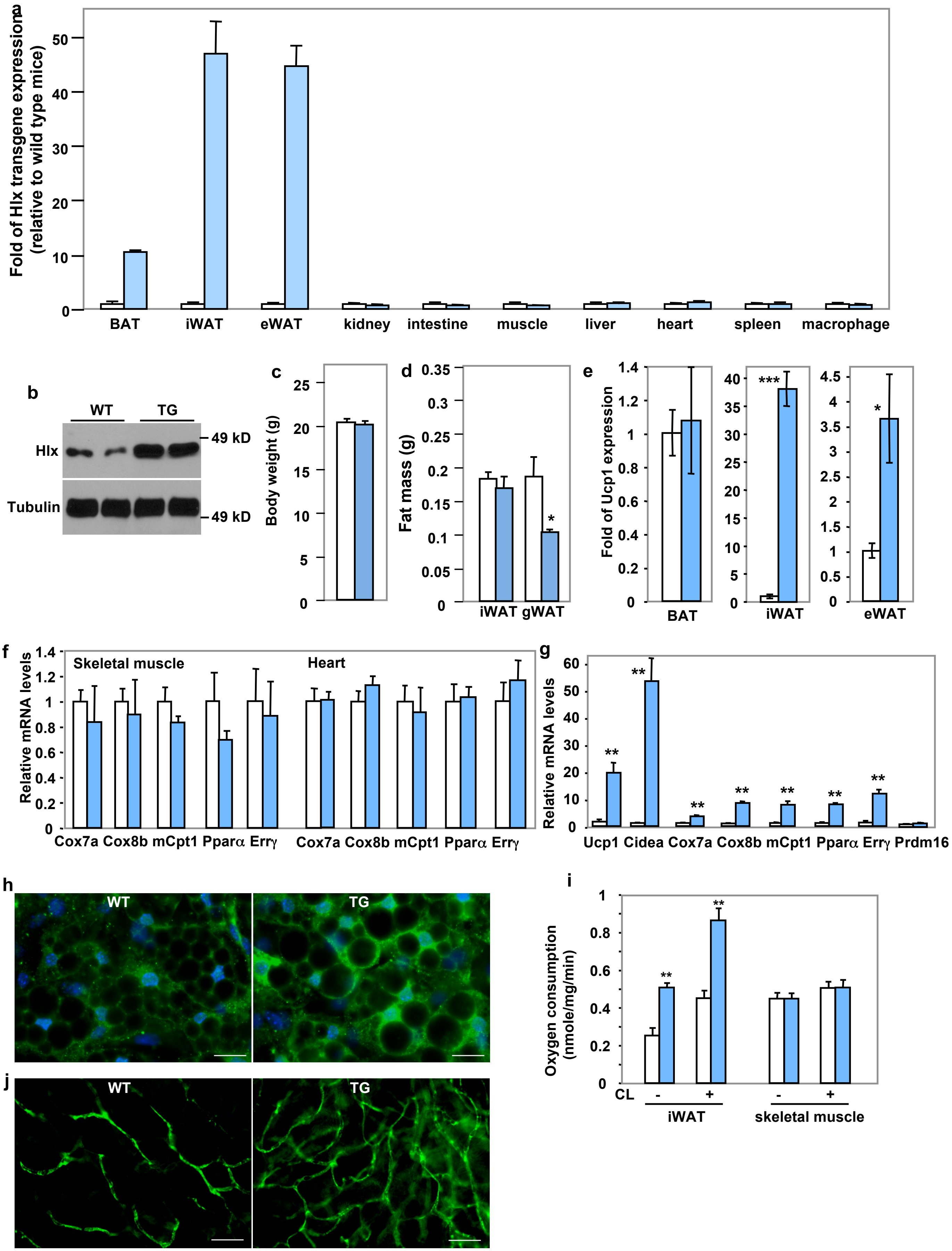
a
Fold of Hlx transgene expression (relative to wild type mice)
BAT
iWAT
eWAT
kidney
intestine
muscle
liver
heart
spleen
macrophage
b
WT
TG
Hlx
Tubulin
49 kD
49 kD
c
Body weight (g)
d
Fat mass (g)
*
iWAT gWAT
e
Fold of Ucp1 expression
***
*
BAT
iWAT
eWAT
f
Relative mRNA levels
Skeletal muscle
Heart
Cox7a Cox8b mCpt1 Pparα Errγ
Cox7a Cox8b mCpt1 Pparα Errγ
g
Relative mRNA levels
**
Ucp1 Cidea Cox7a Cox8b mCpt1 Pparα Errγ Prdm16
h
WT
TG
i
Oxygen consumption (nmole/mg/min)
**
CL
-
+
iWAT
skeletal muscle
j
WT
TG

Supplementary Figure 4. iWAT browning of Hlx transgenic mice.

(a) Fold of Hlx mRNA expression in various tissues of transgenic mice compared with wild type mice (n=3 mice $group^{-1}$). **(b)** Hlx protein expression in eWAT of Hlx transgenic mice and littermate controls. **(c)** Body weights of 12-week-old female Hlx mice (n=7 mice $group^{-1}$) on normal chow diet. **(d)** Fat mass of mice shown in **(c)**. **(e)** Fold of Ucp1 mRNA expression in different fat depots (n=4 mice $group^{-1}$). **(f)** Gene expression in skeletal muscle and heart (n=4 mice $group^{-1}$). **(g)** Gene expression in primary iWAT adipocytes (n=4 mice $group^{-1}$). **(h)** Immunostaining of Tom20 in primary iWAT adipocytes. Shown are representative images of three mice per genotype. Blue, DAPI; Green, Tom20. Scale bar, 200 µm. **(i)** Mice (n=4 mice $group^{-1}$) were intraperitoneally injected with CL-316243 for 3 days or untreated. iWAT and skeletal muscle were used for oxygen consumption assay. **(j)** Fluorescein labeled Lectin I was tail vein injected into mice. iWAT was isolated to visualize blood vessels. Shown are representative images of three mice per genotype. Scale bar, 200 µm.

White bar, wild type; Blue bar, transgenic mice. All error bars represent s.e.m. Two-tailed unpaired Student’s t-test was performed. *p<0.05; **p<0.01.

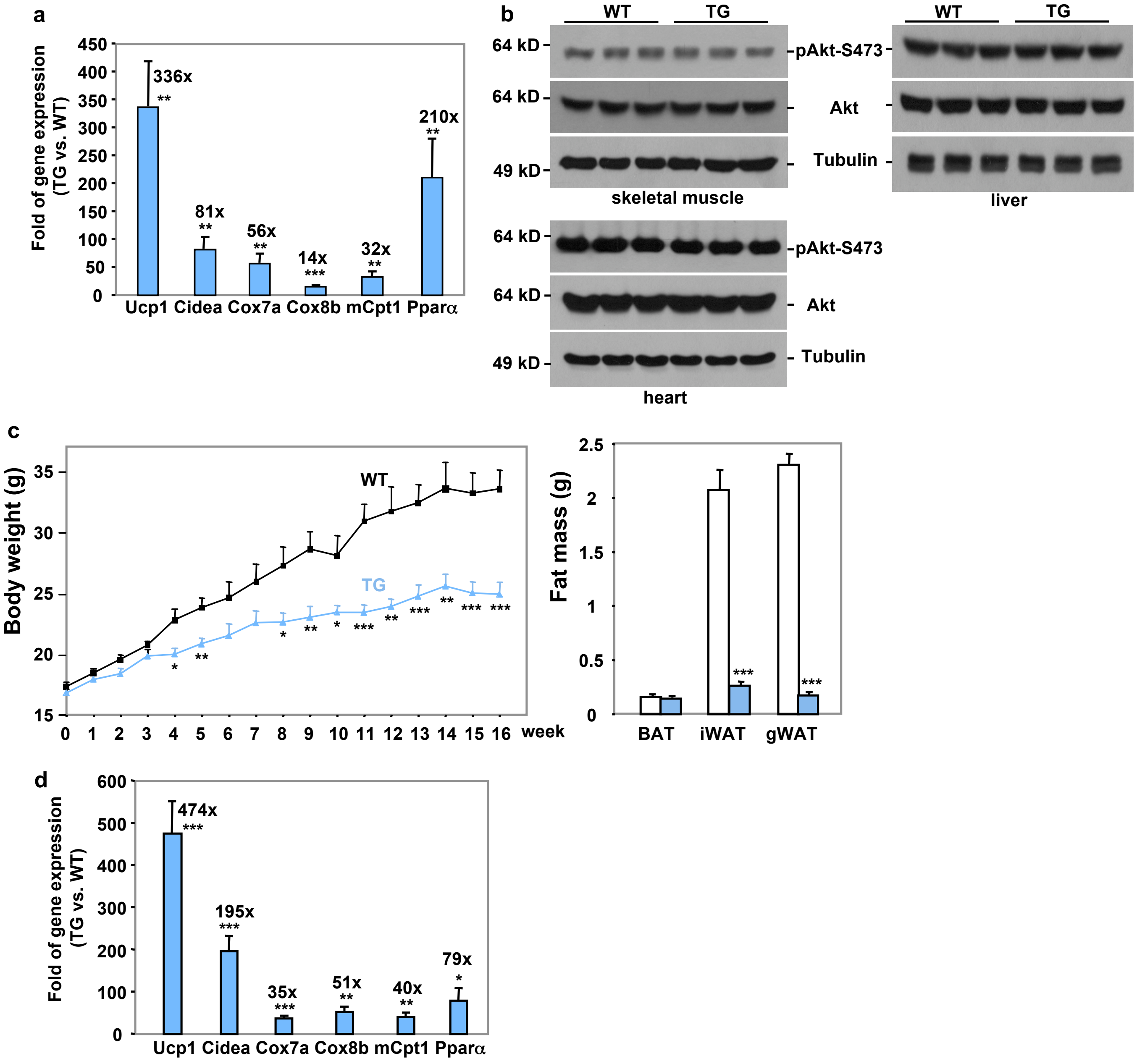

Supplementary Figure 5. Hlx transgenic mice are protected from obesity.

(a) Three-month-old male Hlx transgenic mice and littermate controls (n=4 group^{-1}) were placed at 4°C for 6 hr. Gene expression in iWAT was analyzed and presented as fold. **(b)** Phosphorylation level of Akt at residue S473 in skeletal muscle, liver and heart. **(c)** Body weights and fat mass of female mice (n=7-8 group^{-1}) on a high fat diet for 16 weeks. **(d)** Gene expression in iWAT of mice in **(c)** was analyzed and presented as fold.

All error bars represent s.e.m. Two-tailed unpaired Student's t-test was performed. *p<0.05; **p<0.01; ***p<0.001.

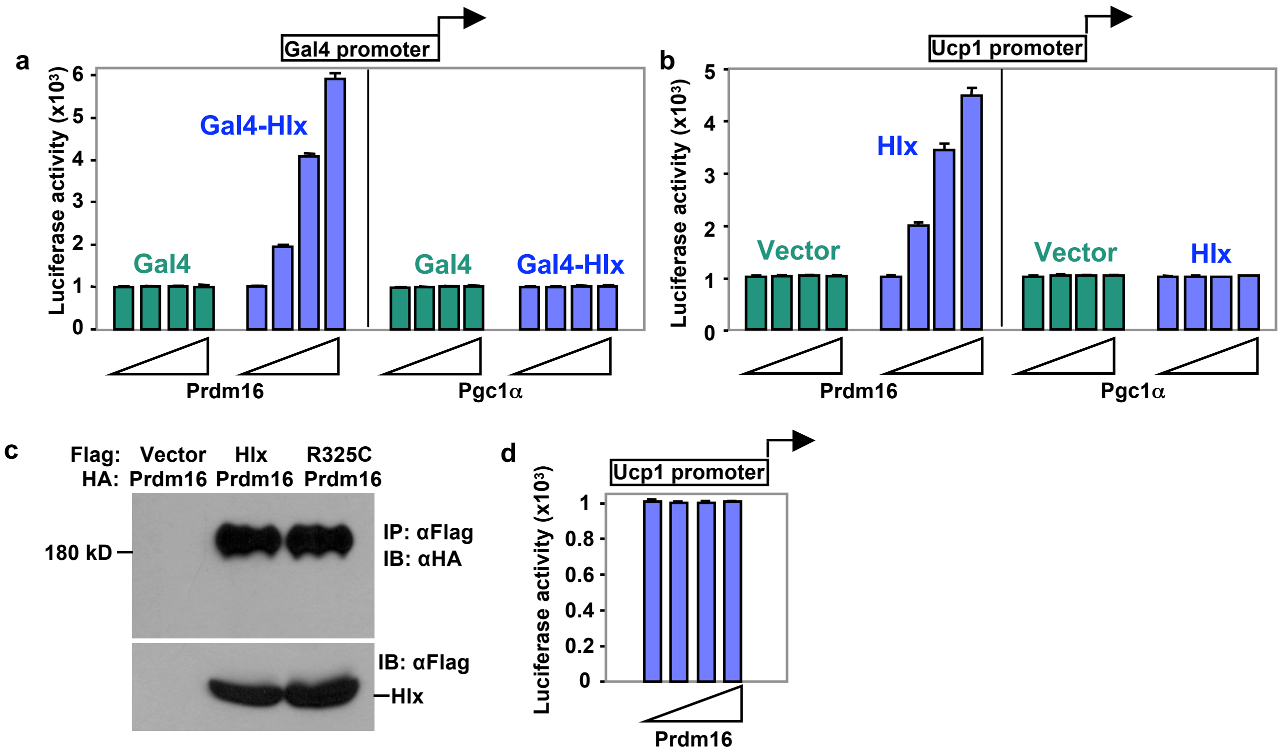

e

	Prdm16 peak	Hlx-binding motif position	Motif sequence
Ucp1 promoter	Chr8: 83289133-83289511	Chr8: 83289199-83289208	TATTTTATTG
	Chr8: 83287563-83288020	Chr8: 83287518-83287527	TTTTAAAATA
Cidea promoter	Chr18: 67343135-67343918	Chr18: 67343265-67343274	TTATTAATGG
Pparα promoter	Chr15: 85732042-85732585	Chr15: 85732111-85732120	TTATCAAAAA
Errγ promoter	Chr1: 187608508-187609053	Chr1: 187608842-187608851	TATTAAAATG
	Chr1: 187608508-187609053	Chr1: 187608952-187608961	TCATTTGTTA

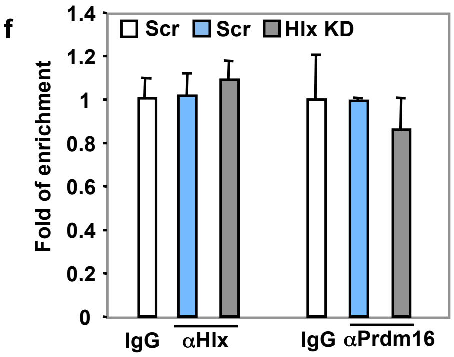

Supplementary Figure 6. Prdm16 is a co-activator of Hlx.

(a) Transcriptional activity of Gal4-Hlx fusion protein on Gal4 promoter in the presence of increased amounts of Prdm16 or Pgc1α (n=3). **(b)** Transcriptional activity of Hlx on the 3.1-kb Ucp1 promoter in the presence of increased amounts of Prdm16 or Pgc1α (n=3). **(c)** Flag tagged Hlx mutant R325C and HA tagged Prdm16 were co-transfected into HEK293 cells. Immunoblot with an HA antibody after immunoprecipitation with a Flag antibody. **(d)** Transcriptional activity of Hlx mutant R325C on the 3.1-kb Ucp1 promoter in the presence of increased amounts of Prdm16 (n=3). **(e)** Shown are genomic coordinates (mm10) and sequences of Hlx-binding motifs located within the Prdm16 peaks at the Promoters of indicated genes. **(f)** ChIP-qPCR analysis of Hlx and Prdm16 association with the aP2 promoter in brown adipocytes with or without Hlx knockdown. Data represent average of two experiments.

All error bars represent s.e.m.

Supplementary Figure 7. Uncropped Western blots

Fig.1b Hlx

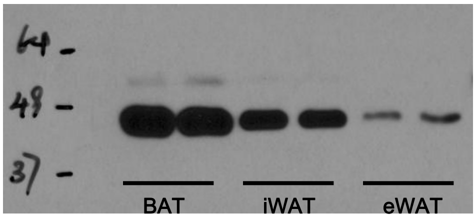

Fig.1b Tubulin

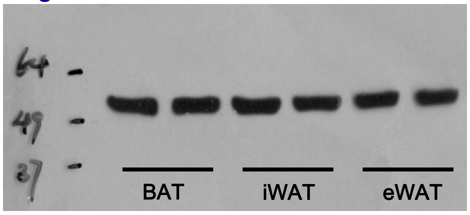

Fig.1c Hlx

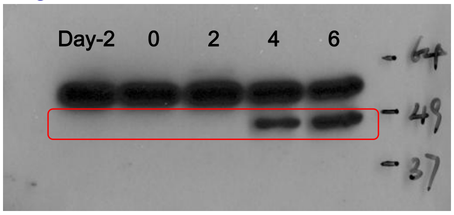

Fig.1c Tubulin

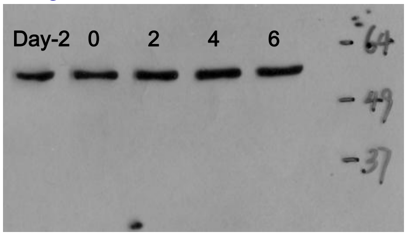

Fig.1e Hlx

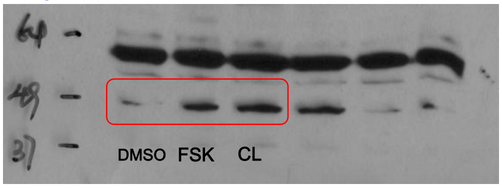

Fig.1e Ucp1

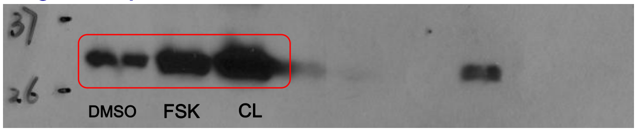

Fig.1e Tubulin

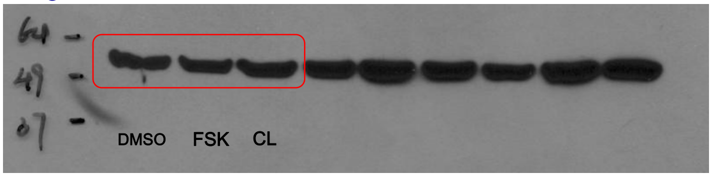

Fig.1f Hlx

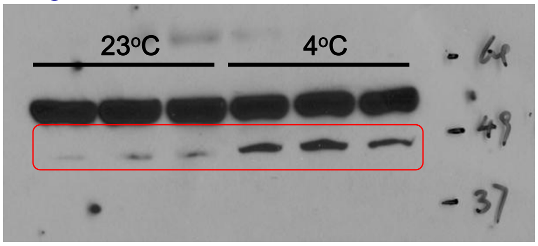

Fig.1f Tubulin

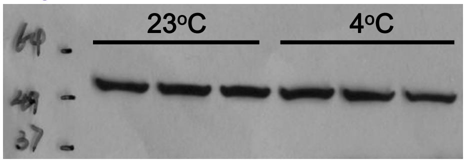

Fig.1g Hlx

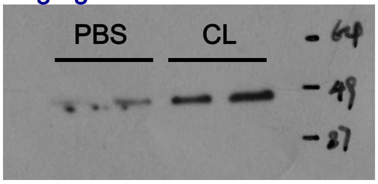

Fig.1g Tubulin

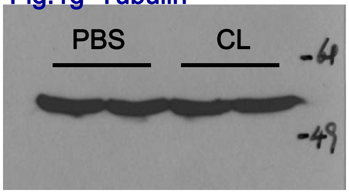

Fig.1h Hlx

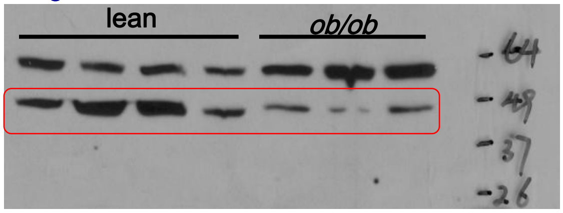

Fig.1h Tubulin

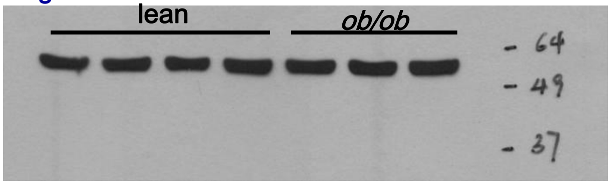

Supplementary Figure 7 (cont). Uncropped Western blots

Fig.1l Hlx

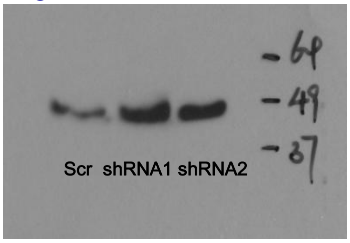

Fig.1l Tubulin

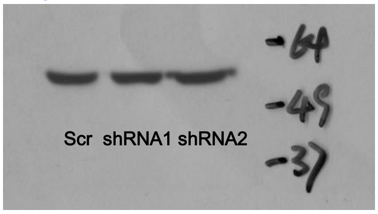

Fig. 2a Hlx

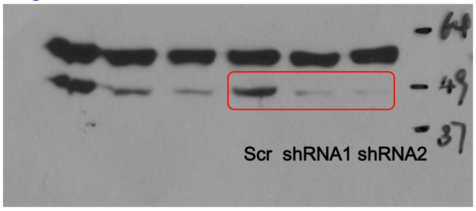

Fig. 2a Tubulin

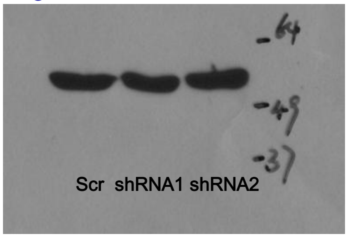

Fig. 2c Tubulin

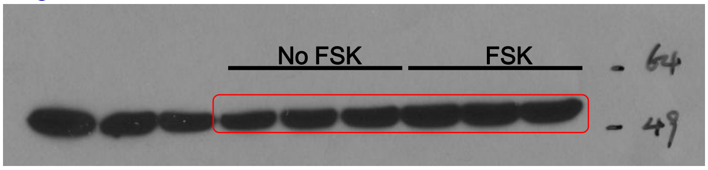

Scr shRNA1 shRNA2 Scr shRNA1 shRNA2

Fig. 2c Ucp1

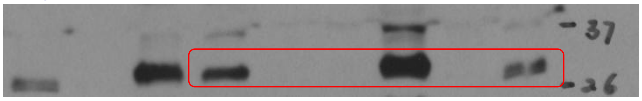

Fig. 2f Hlx

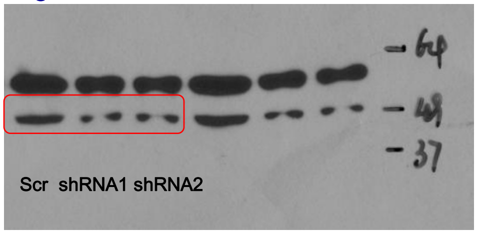

Fig. 2f Tubulin

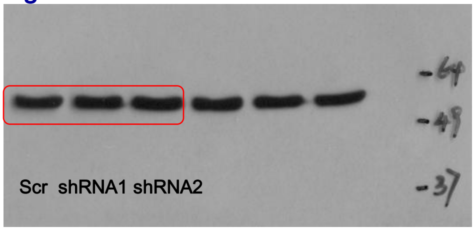

Fig. 3a Ucp1

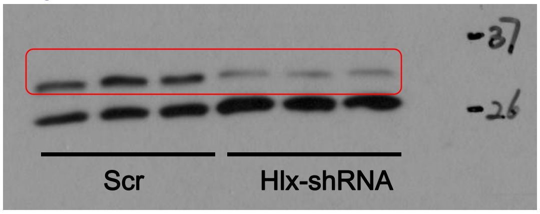

Fig. 3a Hlx

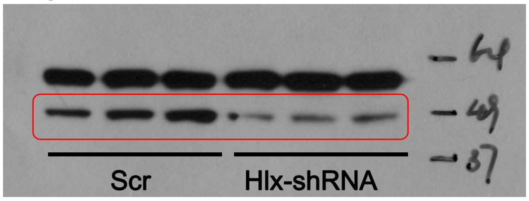

Fig. 3a Tubulin

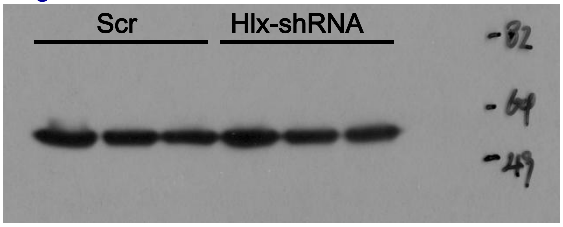

Supplementary Figure 7 (cont). Uncropped Western blots

Fig. 3e Ucp1

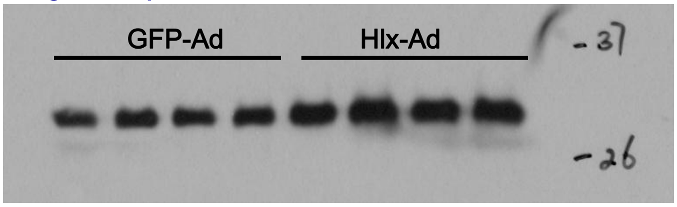

Fig. 3e Hlx

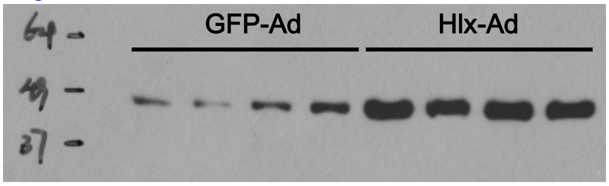

Fig. 3e Tubulin

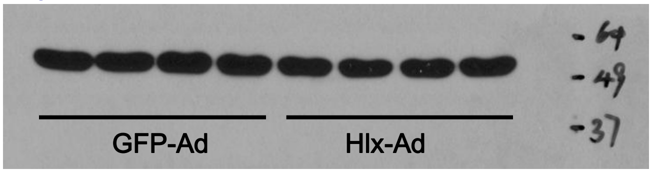

Fig. 4a Ucp1

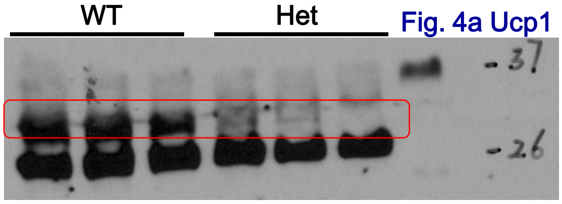

Fig. 4a Hlx

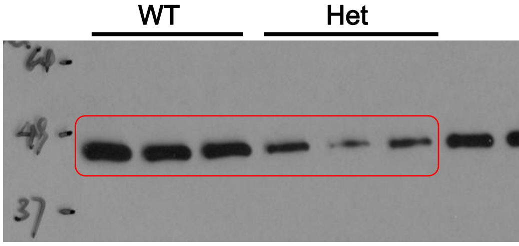

Fig. 4a Tubulin

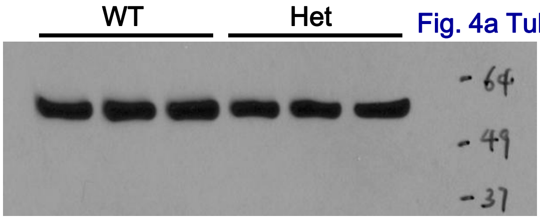

Fig. 4j pAkt-S473

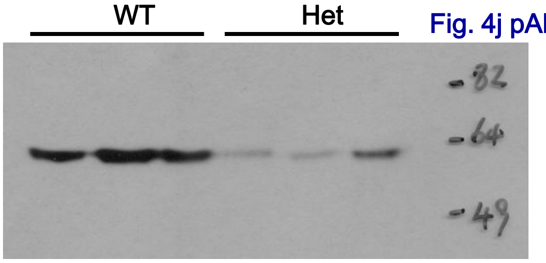

Fig. 4j Total Akt

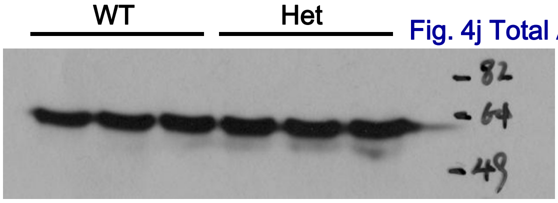

Fig. 4j Tubulin

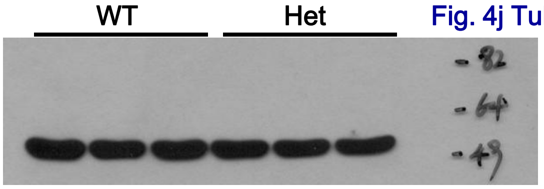

Supplementary Figure 7 (cont). Uncropped Western blots

Fig. 5a Hlx

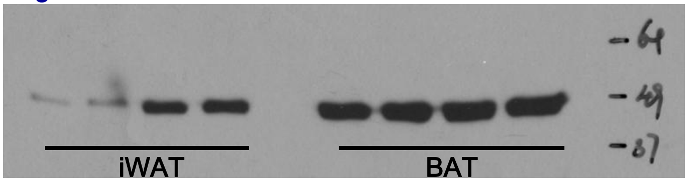

Fig. 5a Tubulin

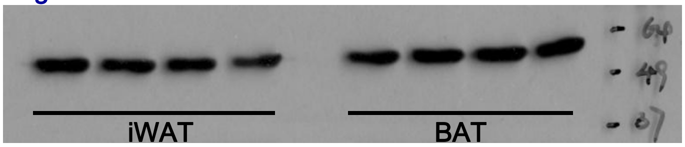

Fig. 5b Hlx

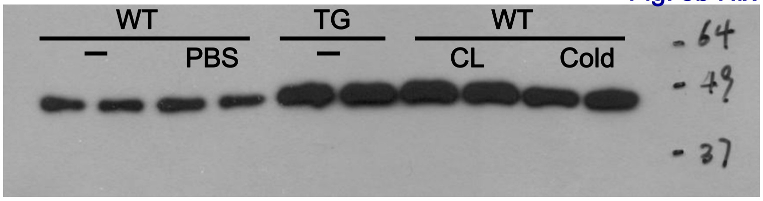

Fig. 5b Tubulin

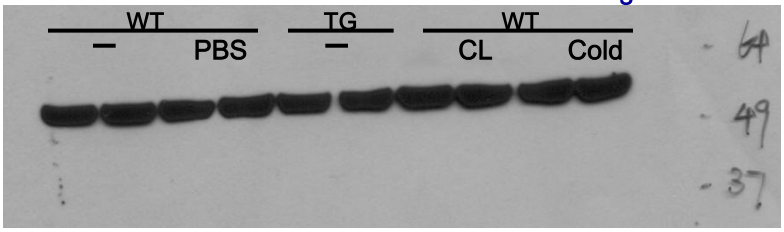

Fig. 6c pAkt-S473

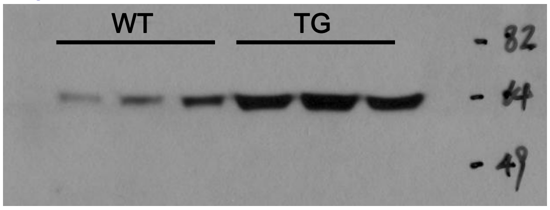

Fig. 6c Total Akt

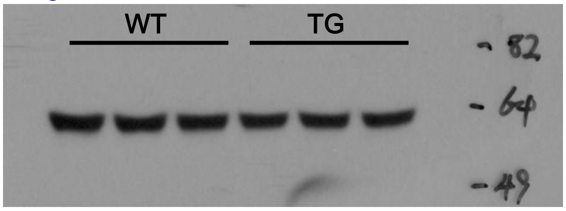

Fig. 6c Tubulin

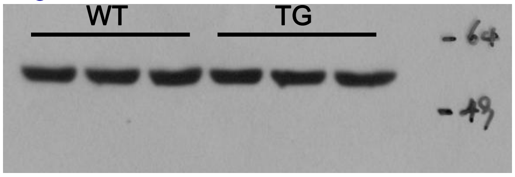

Supplementary Figure 7. Uncropped Western blots

Flag: Vector Hlx Hlx
HA: Prdm16 Prdm16 PGC1α

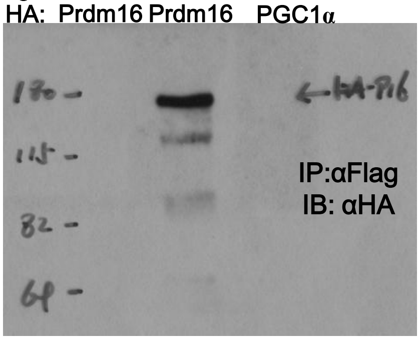

Fig. 7c HA-Prdm16

Vector Hlx Hlx
Prdm16 Prdm16 PGC1α IB: αHA

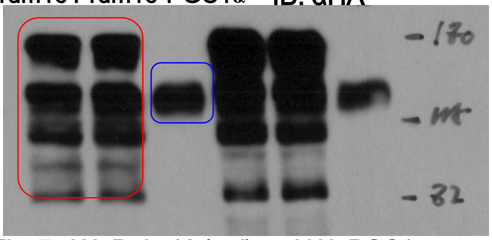

Fig. 7c HA-Prdm16 (red) and HA-PGC1α (Blue)

IB: αFlag Vector Hlx Hlx
Prdm16 Prdm16 PGC1α

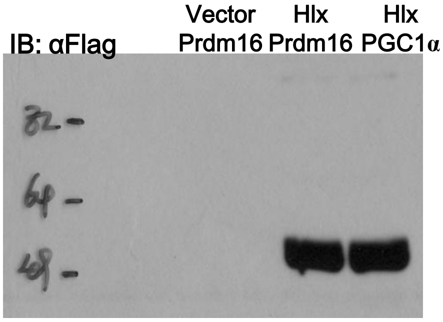

Fig. 7c Flag-Hlx

IP: IgG αHlx

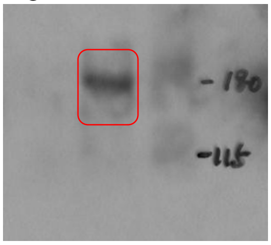

Fig. 7d IB: Prdm16

Input: IgG αHlx

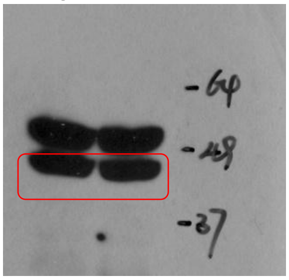

Fig. 7d Hlx

Input: IgG αPrdm16

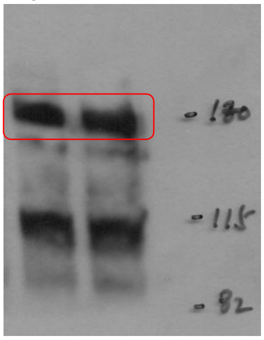

Fig. 7d Prdm16
